# Gaussian and Non-Gaussian Solvent Density Fluctuations within Solute Cavities in a Water-like Solvent

**DOI:** 10.1021/acs.jctc.3c00387

**Published:** 2023-07-12

**Authors:** Henry S. Ashbaugh

**Affiliations:** Tulane University, Chemical and Biomolecular Engineering, New Orleans, Louisiana 70118, United States

## Abstract

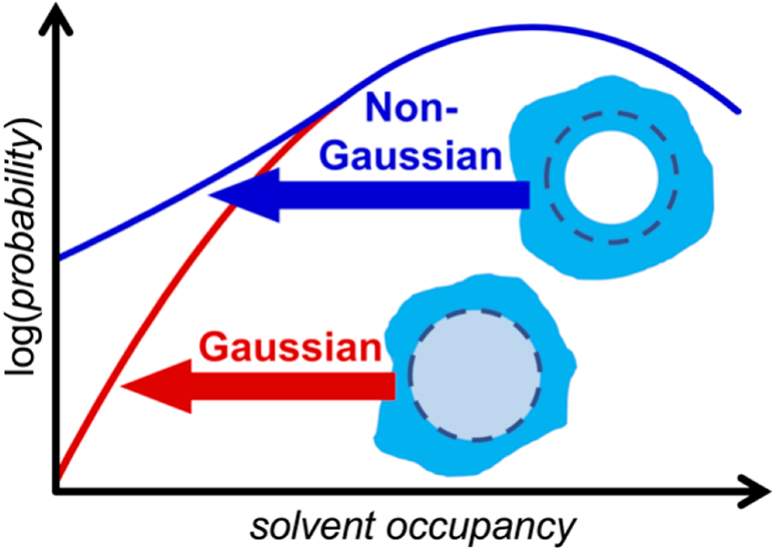

We report a Monte Carlo simulation study of length-scale-dependent density fluctuations in cavities in the coarse-grained mW representation of water at ambient conditions. Specifically, we use a combination of test particle insertion and umbrella sampling techniques to examine the full range of water occupation states in spherical cavities up to 6.3 Å radius in water. As has previously been observed, water density fluctuations are found to be effectively Gaussian in nature for atomic-scale cavities, but as the cavities get larger, they exhibit a non-Gaussian “fat-tail” distribution for lower occupancy states. We introduce a new statistical thermodynamic approach to analyze non-Gaussian fluctuations based on the radial distribution of waters about cavities with varying numbers of waters within its boundaries. It is shown that the onset of these non-Gaussian fluctuations is a result of the formation of a bubble within the cavity as it is emptied, which is accompanied by the adsorption of waters onto its interior surface. We revisit a theoretical framework we previously introduced to describe Gaussian fluctuations within cavities to incorporate bubble formation by including surface tension contributions. This modified theory accurately describes density fluctuations within both atomic and meso-scale cavities. Moreover, the theory predicts the transition from Gaussian to non-Gaussian fluctuations at a specific cavity occupancy, in excellent agreement with simulation observations.

## 1 Introduction

Water plays a central role in self-assembly processes in aqueous solution, including detergent assembly into micelles, folding of proteins, and formation of larger biomolecular structures. The hydrophobic effect, the limited solubility of oily species in water, provides the impetus for the nonspecific aggregation and assembly of the nonpolar constituents of these molecules.^1−3^ The thermodynamic signatures of hydrophobically driven assembly, however, depend on the size and curvature of the aggregating moieties.^4−6^ On the molecular-scale, the dissolution of nonpolar gases and alkanes in water at room temperature is enthalpically favorable but dominated by a large negative entropy that opposes hydration. As the size of the hydrophobic groups increases, the roles of entropy and enthalpy are reversed, and hydration is opposed by a dominant positive enthalpy and favored by a smaller positive entropy. This switch in the signatures of hydrophobic hydration is illustrated by the work of Li and Walker^7^ who observed from experiments of extending polymers into water using an atomic force microscope that the temperature dependence of the pulling force shifted from entropically toward enthalpically unfavored as the size of the nonpolar side chains increased. These observations suggest changes in the hydration mechanism with nonpolar solute size. To gain insight into these processes, many studies have focused on model solutes to isolate hydrophobic effects from competing interactions, like dispersion and electrostatic forces. Molecular simulations are well suited for this task since individual interactions can be turned off while retaining molecular realism.

The standard approach to describe the hydration of nonpolar solutes is to divide the process into two steps.^8^ First, an empty cavity the size and shape of the excluded volume of the solute is created in solution. Second, the attractive interactions between water and the solute placed within the cavity are turned on. For atomic-scale solutes, the characteristic thermodynamics of hydrophobic hydration (e.g., a large negative entropy of hydration) are embedded within the excluded volume contribution to the hydration process, while the attractive contribution can be treated perturbatively. As such, the focus of many studies of hydrophobic hydration has focused solely on the excluded volume, or cavity, contribution to the free energy. The free energy of solvating an empty cavity-like solute is directly related to the probability of finding an empty cavity the size and shape of the solute as
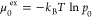
1where *k*_B_*T* is the product of Boltzmann’s constant and the absolute temperature, *p*_0_ is the probability of finding an empty cavity devoid of water within the bulk solvent, and μ_0_^ex^ is the free energy of hydrating the empty cavity, i.e., its excess chemical potential of hydration. One approach to evaluate the excess chemical potential, scaled-particle theory (SPT), focuses on the process of growing empty spherical cavities in solution from nothing up to the desired radius.^9^ The original implementation of SPT only utilized water’s density and its effective hard sphere diameter to describe the hydration process.^10,11^ In the early 1970s, however, Stillinger pointed out that this version of SPT does not account for the structure of liquid water and incorrectly predicts the temperature dependence of water’s liquid–vapor surface tension.^12^ He subsequently provided an empirically corrected SPT utilizing water’s experimental radial distribution function determined from X-ray scattering^13,14^ and the known surface tension of water. Several years later, Ashbaugh and Pratt expanded Stillinger’s approach to incorporate many-body correlations into SPT by using multibody information from molecular simulations.^15−17^ These theories helped illuminate the thermodynamic distinction between molecular-scale hydrophobic hydration, which is opposed by a dominant negative hydration entropy, and meso/macroscale hydrophobic hydration, which is opposed by a dominant positive hydration enthalpy. The distinction between molecular-scale and meso-scale hydration is described by a crossover length,^4,18,19^ which itself is temperature dependent.^17^

In the mid-1990s, Hummer et al.^20^ provided and alternate view of cavity hydration following information theory, where, rather than focusing only on empty cavities, all possible occupancy states are considered, *p*_*n*_ (*n* is the number of solvents within the cavity). They demonstrated that for atomic-scale cavities the *p*_*n*_ distribution is effectively Gaussian in water. While the mean number of solvent molecules in the cavity depends solely on the density of water and the solute’s volume, evaluation of the variance in the distribution requires knowledge of water’s radial distribution function. The information theory description of hydration was subsequently applied to analyze the thermodynamics of hydrophobic hydration,^20,21^ the observation of entropy convergence at elevated temperatures,^22^ and the pressure induced denaturation of proteins.^23^ More recently, Ashbaugh, Vats, and Garde^24^ demonstrated that for state points far from the critical point, the solute-size dependence of the variance of the *p*_*n*_ distribution could be approximated over all size scales using a simple analytical form, referred to as interpolated Gaussian fluctuation theory (IGFT). This theory only requires information on water’s density, compressibility, and effective diameter but not its radial distribution function. IGFT was shown to accurately predict the characteristic thermodynamics of atomic-scale hydrophobic hydration up to 300 °C. Moreover, this theory also predicted that in the supercooled regime, the hydration heat capacity could also reverse its sign from positive to negative in agreement with previous simulation observations.^25−27^

Despite its success at addressing atomic-scale hydrophobic hydration, the Gaussian approximation for solvent density fluctuations breaks down as the solute radius increases much beyond that of xenon. This was perhaps first demonstrated by Huang and Chandler^28^ for the emptying of a Lennard-Jones liquid from cavities significantly larger than the solvent. Notably, they observed that while the *p*_*n*_ distribution is Gaussian for cavity occupancies near the mean, for cavities twice the solvent’s diameter and larger the distribution exhibits a “fat-tail” distribution with lower occupancies being more favorable than anticipated based on Gaussian predictions. They attributed this non-Gaussian tail to the onset of drying as a result of a bubble forming within the cavity as it is emptied. It has subsequently been demonstrated that the onset of non-Gaussian density fluctuations in water between the hydrophobic faces of biomolecules impacts their interactions with each other and interfaces.^29,30^ Moreover, water density fluctuations within hydrophobic pockets can tilt so that they spontaneously dewet, favoring the binding of nonpolar guests to these surfaces.^31^

In a recent paper, Sinha et al.^32^ examined the process of hydrating large spherical and nonspherical solutes in SPC/E water to determine the point at which water density fluctuations within solute cavities cross over from Gaussian to non-Gaussian behavior. In this analysis, we introduced a theoretical approach utilizing the known interfacial properties of water to describe the onset of bubble formation within the cavity to describe non-Gaussian density fluctuations. That theoretical approach, however, was introduced in a cursory manner and was not fully developed. Here we revisit this problem to provide a clearer justification for the proposed changes in the mechanism of cavity emptying in solutions, reporting new molecular simulations and theoretical results for the process of emptying solute volumes in water to gain insights into the role of solvent density fluctuations in the thermodynamics of hydrophobic hydration. Water is modeled here using the coarse-grained mW model developed by Molinero.^33^ This model neglects water’s hydrogens, capturing the effect of directional hydrogen-bonding using a three-body potential proposed by Stillinger and Weber^34^ that is shorter-ranged than more traditional representations of water, making it computationally more expedient. More importantly for the work reported here, it has been shown to capture many of the peculiar thermodynamics of hydrophobic hydration.^35^ In this work, we use simulations to determine the free energies of removing individual waters from cavities of varying radius to generate a hard-sphere solute in water, a model solute for understanding hydrophobic hydration. A new statistical mechanical approach is developed to evaluate the work of emptying the cavities based on the structure of water about partially filled and empty cavities, thereby providing new insights into the emptying process. In the final part of the paper, we extend IGFT to incorporate non-Gaussian fluctuations that result from interfacial effects that arise as the solute grows in size.

## 2 Theory

### 2.1 Water Partitioning between a Cavity Interior and the Bulk Solvent

While the empty cavity (*n* = 0) is most relevant for evaluation of the solvation free energy of a hard sphere solute as embodied by eq 1, as shown here, the intermediate *n* provides significant information on the mechanism by which the volume is emptied. In particular, this chemical potential for creating an empty cavity can be readily extended to consider the process of finding a cavity with *n* water in it as μ_*n*_^ex^ = −*k*_B_*T* ln *p*_*n*_. The process of removing solvent molecules from a cavity to create a hard-sphere solute can be broken down into a series of steps in which the solvent molecules are removed one-by-one from the volume. In this section, we consider the emptying process to construct a description of hard sphere solvation based on the packing of solvent molecules both inside and outside the cavity.

To begin, we consider the thermodynamic equilibrium between waters inside and outside of a cavity in solution. The canonical partition function of a system of *N* total water molecules with *n* waters confined within the boundaries of the cavity, υ, and *N – n* waters outside the cavity is
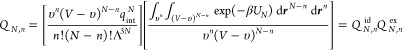
2where *V* is the total system volume, Λ is the thermal de Broglie wavelength, *q*_int_ is the internal partition function for an individual water molecule associated with vibrational and rotational degrees of freedom, *U*_*N*_ is the system potential energy, ***r*** is the position vector of an individual molecule, and β = 1/*k*_B_*T*. Orientational degrees of freedom are neglected in the integral above, but this does not change the results of this analysis. The terms in the first set of square brackets correspond to the ideal gas contribution to the partition function, *Q*_*N,n*_^id^, while the second set of square brackets corresponds to the excess contribution to the partition function, *Q*_*N,n*_^ex^, resulting from intermolecular interactions. The ratio of probabilities of observing *n* + 1 and *n* molecules within the cavity is related to the ratio of their partition functions as
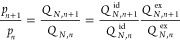
3In the thermodynamic limit (*N* → ∞ and *V* → ∞) the ratio of the ideal gas partition functions is
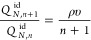
4where ρ = *N*/*V* is the total solvent number density. The ratio of the excess partition functions is
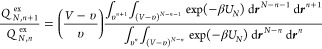
5This ratio can be re-expressed as
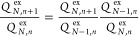
6a
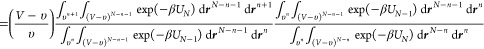
6b

The energy of the *N* molecule system can be divided into *N* - 1 and 1 molecules as
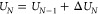
7It follows that the first ratio of integrals in eq 6b can be expressed as
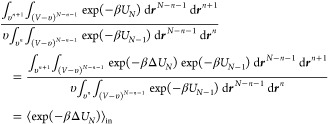
8which corresponds to the Boltzmann weighting of the mean excess chemical potential of a particle randomly inserted into the cavity. Similarly, the second ratio of integrals in eq 6b can be expressed as
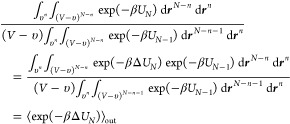
9which corresponds to the Boltzmann weighting of the mean excess chemical potential of a particle randomly inserted outside the cavity. Given that the volume of the solvent outside of the cavity is infinitely greater than that inside the cavity, this is simply the excess chemical potential of the bulk solvent. The ratio of cavity occupation probabilities is subsequently
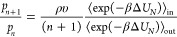
10While the averages in eqs 8–10 are technically for the addition of a water molecule to a system of *N* – 1 waters, in the thermodynamic limit as the system size and number of waters becomes infinite this is indistinguishable for the averages of adding a water molecule to an *N* water system.

While eq 10 could be evaluated from simulation following standard particle insertion techniques, this expression can be more readily evaluated from the occupancy dependent radial distribution functions (RDFs) between the cavity and the solvent, *g*_*n*_(*r*). This collection of RDFs corresponds to the local solvent density as a function of the distance from the center of a cavity with *n* waters residing within it. Associated with each RDF is a function y_*n*_(*r*), also referred to as a cavity correlation function, that corresponds to the Boltzmann weighting of the potential-of-mean force associated with bringing a solvent particle from a position infinitely far away to a distance *r* from the center of the cavity with *n* molecules constrained to reside inside. The cavity correlation function is determined following test particle insertion as
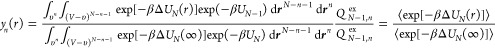
11

The numerator corresponds to the Boltzmann weighting of the excess chemical potential for a water at a distance *r* from the center of the cavity, while the denominator corresponds to the Boltzmann weighting of the excess chemical potential for a water infinitely far away from the center of the cavity, which is equal to the bulk chemical potential in eq 9 (i.e., ⟨exp(−βΔ*U*_*N*_ (∞))⟩ = ⟨exp(−βΔ*U*_*N*_)⟩_out_. For separations outside the cavity, y_*n*_ (*r* > *R*) corresponds to the RDF between the solvent and cavity with *n* molecules within it (i.e., y_*n*_ (*r* > *R*) = *g*_*n*_ (*r* > *R*)). For distances inside the cavity, *n* + 1 molecules now reside within its boundary. While it is tempting to equate y_*n*_ (*r* ≤ *R*) with *g*_*n*__+1_(*r* ≤ *R*), normalization of the radial distribution function negates this equality. Specifically, within the cavity the radial distribution function obeys the condition
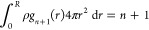
12This suggests, as we shall see below, that the RDF changes discontinuously across the cavity boundary, while *y*_*n*_(*r*) is expected to be a continuous function. We propose that *y*_*n*_(*r*) is related to the radial distribution functions as
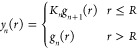
13where *K*_*n*_ is a constant that ensures *y*_*n*_(*r*) is continuous across the cavity’s boundary. The question follows, what is *K*_*n*_?

Following from eq 12, the average value of *g*_*n*__+1_(*r*) inside the spherical cavity (*r* ≤ *R*) is
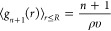
14It follows from eq 13 that the average value of *y*_*n*_(*r*) inside the cavity is
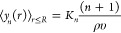
15Following eq 11, the average value of *y*_*n*_(*r*) inside the cavity is also
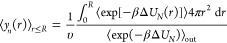
16The mean value of the integral in the numerator over the cavity interior is
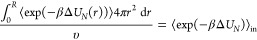
17Combining eqs 10, 16, and 17, it follows that
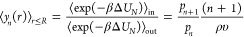
18Comparing eqs 15 and 18 we find the desired result that
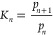
19Thus, the discontinuous change in the solvent RDF across the boundary of the cavity can be related to the work associated with adding a solvent molecule to the cavity already containing *n* molecules.

The derivations above provide a route to determining *p*_*n*_ from molecular simulations, simply from the distribution of solvent molecules in a cavity. Specifically, a cavity may be included within a simulation box to evaluate the *g*_*n*_(*r*) distributions. The resulting *K*_*n*_’s can be determined by enforcing the continuity between *g*_*n*_(*r*) and *g*_*n*__+1_(*r*) at *R* to determine *y*_*n*_(*r*). In practice, over a small range near the boundary, −ln *y*_*n*_(*r*) can be approximated by a quadratic polynomial. As such we fit the function
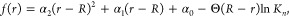
20simultaneously to −ln *g*_*n*__+1_(*r*) for *r* < *R* and −ln *g*_*n*_(*r*) for *r* ≥ *R*. Here Θ(*x*) is the Heaviside function, and α_0_, α_1_, α_2_, and ln *K*_*n*_ are fitting constants. This fitting is performed over only a limited range of *R* ± 1 Å, beyond which the quadratic approximation becomes increasingly inaccurate. Over this limited fitting range, the potential-of-mean force is −*k*_B_*T* ln *y*_*n*_(*r*) = α_2_(*r* – *R*)^2^ + α_1_(*r* – *R*) + α_0_. Once the set of *K*_*n*_’s across all potential cavity occupation states is determined, the full *p*_*n*_ distribution is given as
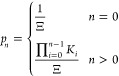
21awhere
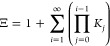
21b

While the cavity does exhibit correlations with the solvent that depend on *n*, on average, the cavity itself has no correlations with the solvent. As such the average RDF between the cavity and solvent is expected to be
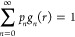
22over all separations from inside to outside the cavity, reflecting the passive nature of the cavity.

To demonstrate the validity of this approach for obtaining the solvent occupancy distribution within a cavity, we consider the ideal gas for which *p*_*n*_ is known analytically. In the ideal gas where no interactions between molecules are felt, the cavity correlation function between a gas particle and a cavity with *n* particles within its interior is
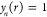
23

As a result of the normalization condition inside the cavity (eq 14), however, the *n* dependent RDFs are given as
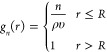
24Comparing eqs 18, 23, and 24, it follows that
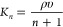
25

Substituting eq 25 into eq 21, we obtain
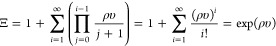
26aand
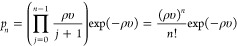
26bThis is the Poisson distribution, the expected probability distribution for a cavity in an ideal gas. Further, averaging the *n* dependent RDFs within the cavity (*r* ≤ *R*) yields
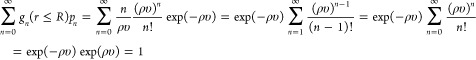
27

Since the Poisson distribution is normalized, it follows that the average of the radial distribution functions outside the cavity is 1 as well. The average of the cavity radial distribution functions in the ideal gas subsequently obeys eq 22.

### 2.2 Interpolated Gaussian Fluctuation Theory

Here we highlight the key equations underlying the IGFT description of solvent occupation probabilities in atomic-sized cavities in water. A more complete development of this theory can be found in ref (24). Following information theory utilizing only information on the second moment of solvent density fluctuations,^20^ the solvent occupancy probability distribution within an atomic-sized cavity is expected to be Gaussian in nature. Assuming that this size range *n* can be approximated as a continuous variable that spans from −∞ to ∞, the probability of observing *n* solvent molecules within the cavity can be expressed using the normalized Gaussian distribution^21,22^
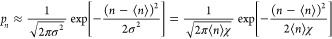
28where ⟨*n*⟩ = *ρυ* is the mean number of solvent molecules within the cavity, σ^2^ is the variance of the distribution, and χ = σ^2^/⟨*n*⟩ is the normalized variance. The excess chemical potential associated with creating a cavity with *n* solvent molecules within its boundaries is subsequently
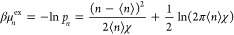
29aIt follows that the hydration free energy of the empty cavity is
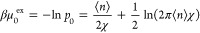
29b

While clearly an approximation, this description of atomic scale solvation has been successfully applied to describe hard sphere solvation in water over a wide range of temperatures.^22,36^

While ⟨*n*⟩ is simply determined from the bulk solvent density and the solute volume, χ is determined from a more complex integral over the solvent–solvent RDF and is not analytical. Nevertheless, considering the microscopic and macroscopic limits on the χ integral, Ashbaugh, Vats, and Garde^24^ proposed an interpolative analytical approximation for χ in spherical cavities as a function of their radius
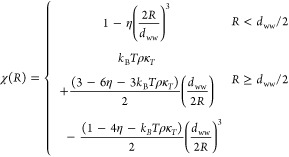
30where *κ*_*T*_ is water’s isothermal compressibility, *d*_ww_ is water’s effective diameter, and η = *πρd*_ww_^3^/6 is the solvent packing fraction. The expressions eqs 28 to 30 are collectively referred to as IGFT. This framework was shown to provide an excellent description of the hydration of hard cavities in water over a wide temperature range and even predicted the unanticipated reversal in the temperature dependence of hydrophobic hydration thermodynamics in the deeply supercooled regime. Interestingly, eq 30 largely relies only on water’s macroscopic equation-of-state, while the structure of water is primarily embodied within *d*_ww_ (assumed here to be 2.65 Å). This diameter has been found to be effectively independent of temperature.^24,25^ The integrated pair-correlation structure of water is incorporated into the theory through its compressibility as determined by Kirkwood-Buff theory,^37^ although this plays a secondary role to *d*_ww_. In previous studies, non-Gaussian fluctuations were found to become more significant as the size of the solute grows.^28,32,38^ As such, IGFT is expected to become increasingly inaccurate for meso-scale and larger solute volumes.

## 3 Molecular Simulations

Monte Carlo (MC) simulations^39^ of mW water^33^ with a single cavity in solution were conducted in the isothermal–isobaric ensemble. The temperature and pressure were set to 25 °C and 1 atm, respectively. Spherical cavity radii of 2.5–6.3 Å in 0.2 Å increments were considered. For cavity radii up to 4.1 Å, simulations were conducted with 700 waters, while 1000 waters were considered for the larger solutes. The cavity, in principle, has no interactions with water, serving to sample solvent density fluctuations within its boundaries. For large volumes, however, the rarity of large density fluctuations ensures that empty or nearly empty cavities are never observed. To sample large scale density fluctuations we applied a biasing harmonic umbrella potential^40^ to restrict the range of cavity occupancies observed during the simulation
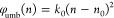
31

Here *k*_0_ is the spring constant, and *n*_0_ is the occupancy for which the umbrella potential is a minimum. The value of *n*_0_ was adjusted from 0 to values well above ⟨*n*⟩ in increments of Δ*n*_0_ = 2 to ensure sampling of the full range of relevant occupancy states. The spring constant was adjusted to sample occupancy states of ±3 on either side of *n*_0_ to ensure overlapping fluctuations between simulations with consecutive values of *n*_0_. As such, *k*_0_ took on values from 2.5 to 9 kJ/mol depending on *n*_0_, with larger *k*_0_ values needed as the cavity is emptied. The full *p*_*n*_ distribution was reconstructed using the weighted histogram analysis method.^41,42^ At least 10^6^ MC passes (where 1 MC pass corresponds to 1 attempted move on each water molecule) were performed for equilibration at each value of *n*_0_, followed by 2.5× 10^7^ passes for evaluation of thermodynamic averages. Volume moves were performed every 5 MC passes. The water and cavity displacements were modified to ensure acceptance of 30% of moves, while the total simulation volume displacement was similarly modified to ensure acceptance of 30% of the attempted changes.

In addition to simulations of an explicit cavity in water, we also performed MC simulations of pure mW water at 25 °C and 1 atm. The cubic simulation cell contained 700 water molecules. In addition to gathering information on the pure water properties (e.g., density and compressibility), we evaluated the excess chemical potentials of atomic-sized solutes using Widom test particle insertion.^43,44^ 10^5^ random test particle insertion attempts were made every tenth MC pass. 10^6^ MC passes were conducted for equilibration, followed by 10^8^ MC passes for the evaluation of thermodynamic averages.

## 4 Results and Discussion

### 4.1 Density Fluctuations within a Spherical Cavity

**Figure 1:**
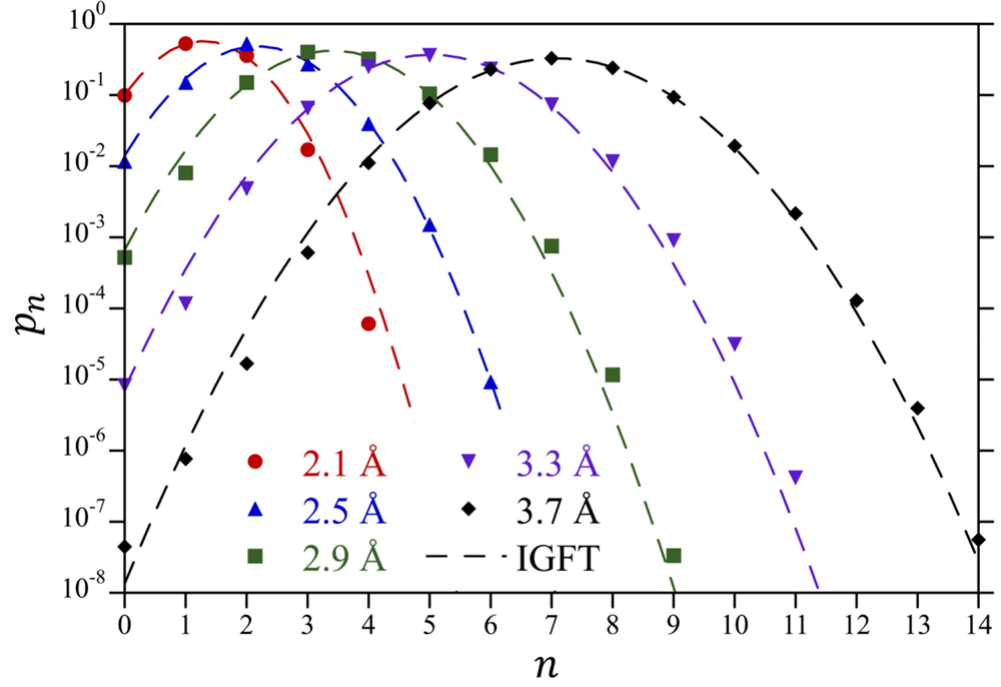
Water occupational probability distributions, *p*_*n*_, within cavities 2.1 Å, 2.5 Å, 2.9 Å, 3.3 Å, and 3.7 Å in radius at 25 °C and 1 atm. Simulation results determined by umbrella sampling are compared against the predictions of IGFT using symbols defined in the figure legend. The simulation errors are smaller than the figure symbols.

Figure 1 illustrates the Gaussian nature of solvent density fluctuations within atomic-scale cavities up to 3.7 Å in radius in mW water at 25 °C and 1 atm. Specifically, when the probability of observing *n* water molecules within the cavity, *p*_*n*_, is plotted on a logarithmic scale as a function of *n*, we find that the distributions effectively assume a parabolic form consistent with a Gaussian distribution. The simulation results are in excellent agreement with the distributions predicted by IGFT, lending confidence to the fidelity of the theory for describing small-scale solute hydration.

**Figure 2:**
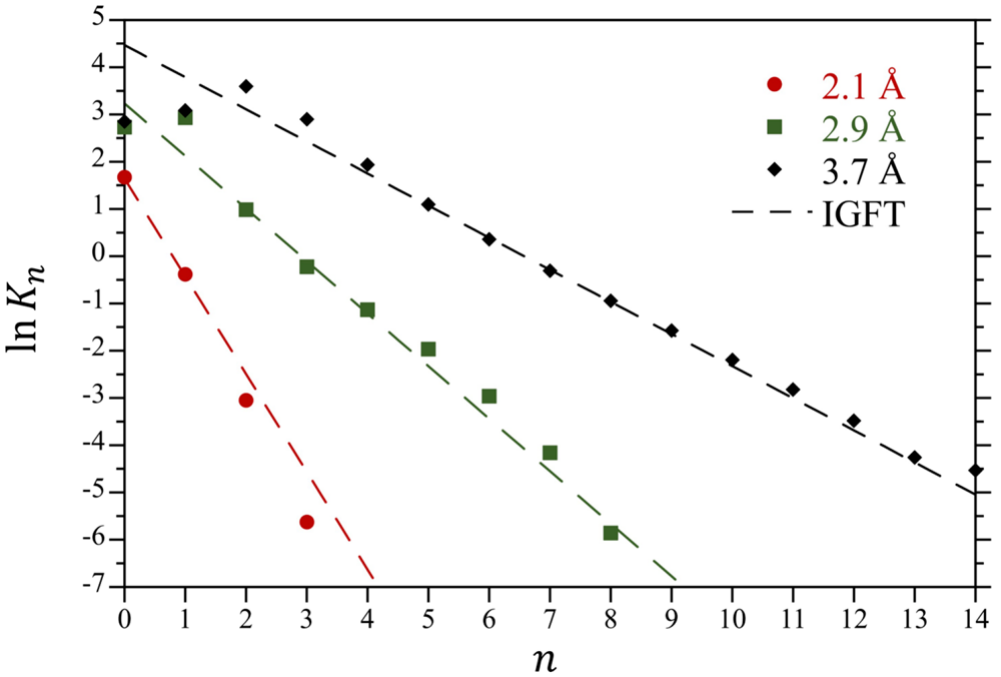
Differential change in the water occupational probability, ln *K*_*n*_ = ln(*p*_*n*__+1_/*p*_*n*_) for the 2.1 Å, 2.9 Å, and 3.7 Å radius cavities at 25 °C and 1 atm. Simulation results determined by umbrella sampling are compared against the predictions of IGFT using symbols defined in the figure legend. The simulation errors are smaller than the figure symbols.

The accuracy of the Gaussian description can be further probed by plotting ln *K*_*n*_ (= ln(*p*_*n*__+1_)/*p*_*n*_)) versus *n* (Figure 2). The expectation is that if the density fluctuations are Gaussian (i.e., ln *p*_*n*_ = *an*^2^ + *bn* + *c*, where *a*, *b*, and *c* are constants), then ln *K*_*n*_ will be a linear function of *n* (i.e., ln *K*_*n*_ = 2*an* + *a* + *b*). While IGFT predicts this linear dependence by construction, the simulation results exhibit deviations from linearity with increasing solute size. The simulation results for each cavity considered in Figure 2 exhibit linear behavior for occupation numbers close to the mean (⟨*n*⟩ = *ρυ* = 1.3, 3.4, and 7.1 for the 2.1 2.9, and 3.7 Å radius cavities, respectively), indicative of Gaussian-like fluctuations near the mean of the distribution that are well described by IGFT. For more extreme fluctuations away from ⟨*n*⟩, however, this does not necessarily hold. Notably for the 3.7 Å radius sphere, the simulations exhibit a slight positive deviation from linearity for *n* = 2 and 3, followed by a drop below the linear prediction for *n* = 0 and 1. Analogous behavior can be observed for the 2.9 Å radius sphere as well, although it is not as dramatic as for the larger volume. The 2.1 Å radius cavity, on the other hand, is accurately described by Gaussian prediction. For these atomic-scale volumes, however, the combination of positive and negative deviations from linearity for ln *K*_*n*_ are largely compensatory, such that the Gaussian predictions for the probability of observing an empty sphere, i.e., *p*_0_, are reasonably accurate up to 3.7 Å (Figure 1).

**Figure 3:**
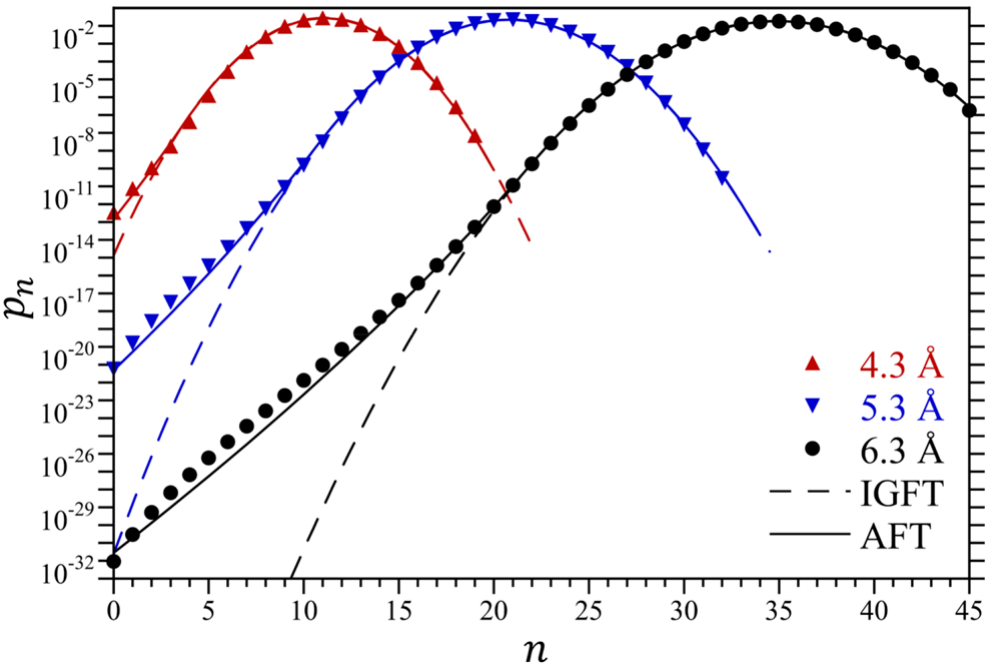
Water occupational probability distributions, *p*_*n*_, within cavities 4.3 Å, 5.3 Å, and 6.3 Å in radius at 25 °C and 1 atm. Simulation results determined by umbrella sampling are compared against the predictions of IGFT and AFT using symbol defined in the figure legend. The simulation errors are smaller than the figure symbols.

The deviations from Gaussian fluctuations are more significant with an increasing cavity size. The occupation probabilities for the 4.3, 5.3, and 6.3 Å radius cavities shown in Figure 3 are parabolic on a logarithmic scale near the maxima in these distributions, and hence are Gaussian close to ⟨*n*⟩. As *n* decreases, however, the probability distributions for each of these volumes exhibit markedly greater probabilities for observing nearly empty cavities than anticipated based on Gaussian fluctuations. As such, the free energy cost associated with observing an empty mesoscopic-sized cavity would be much lower than that predicted following a Gaussian description. This deviation from the Gaussian prediction corresponds to the fat-tail distribution associated with drying phenomena in the context of hydrophobic phenomena.^28,29^

**Figure 4:**
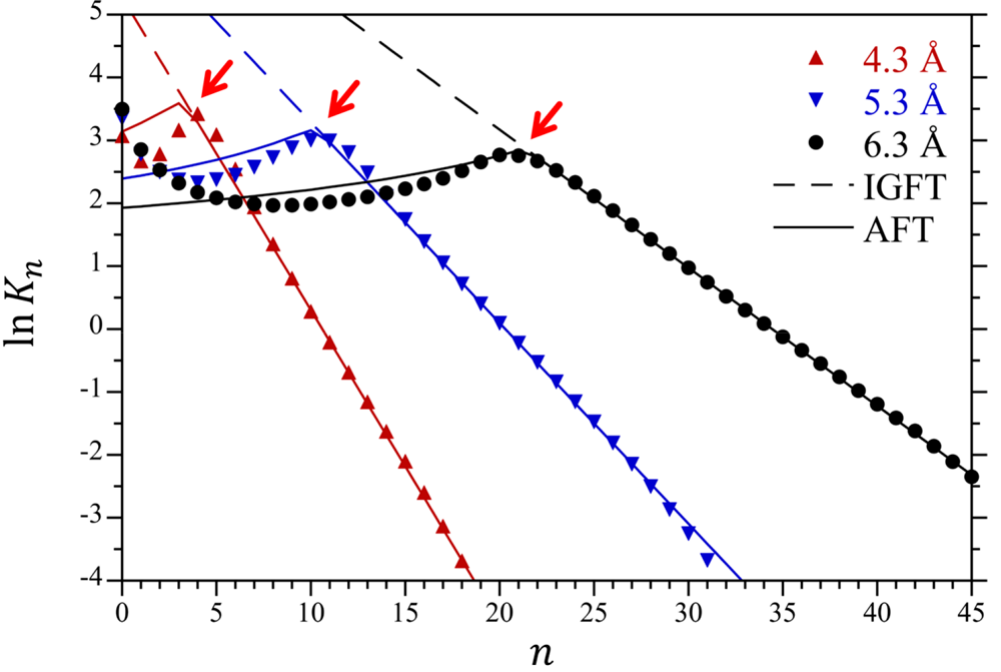
Differential change in the water occupational probability, ln *K*_*n*_ = ln(*p*_*n*__+1_/*p*_*n*_) for the 4.3 Å, 5.3 Å, and 6.3 Å radius cavities at 25 °C and 1 atm. Simulation results determined by umbrella sampling are compared against the predictions of IGFT and AFT using symbols defined in the figure legend. The simulation errors are smaller than the figure symbols.

The breakdown of the Gaussian description for larger volumes is further scrutinized in the plot of ln *K*_*n*_ versus *n* in Figure 4. As above, near the mean occupation number (⟨*n*⟩ = 11.1, 20.8, and 34.9 for the 4.3 5.3, and 6.3 Å spheres, respectively) the simulation results are linear and well described by IGFT. As *n* gets smaller, however, each of these cavities exhibits a dramatic break from linearity. The location of this break depends on the size of the sphere. To a first approximation, ln *K*_*n*_ from *n* = 0 to the break from linearity is (very) roughly constant and equal to ∼2.5, although there is an *n* dependence in this regime. A conclusion that can be drawn is that the crossover from Gaussian to non-Gaussian density fluctuations is not gradual but rather exhibits a marked change as *n* drops, suggestive of a transition in the cavity emptying mechanism.

**Figure 5:**
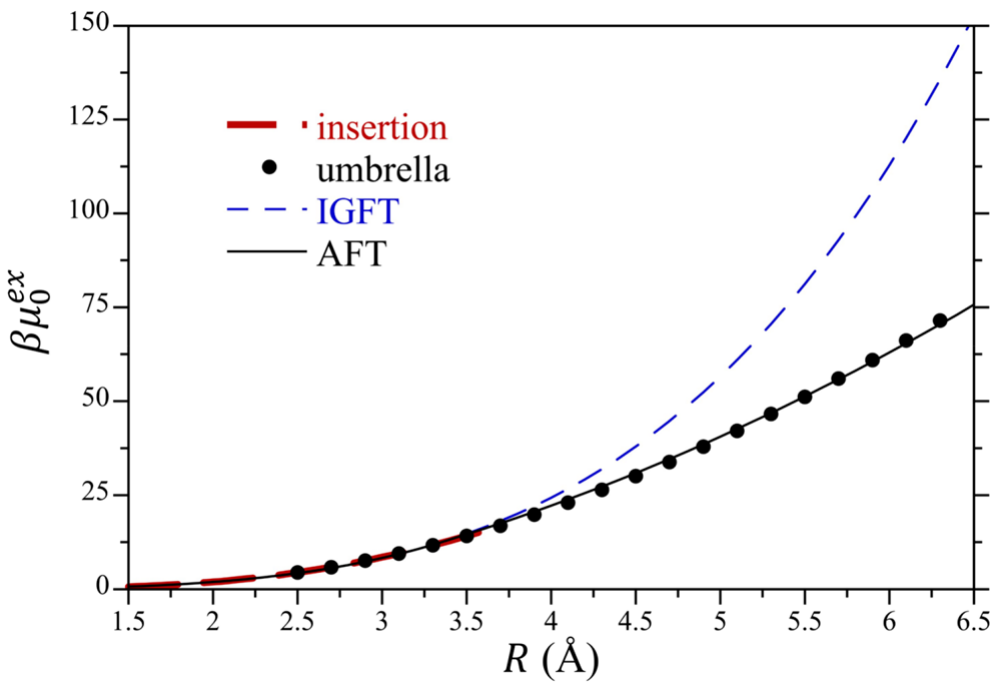
Excess chemical potentials of hard sphere solutes (i.e., empty cavities) in water as a function of their radius at 25 °C and 1 atm. Simulation results determined by test particle insertion and umbrella sampling are compared against the predictions of IGFT (eq 29b) and AFT (eq 44) using the symbols defined in the figure legend. Simulation errors are smaller than the figure symbols.

The differences in the emptying process of the atomic and meso-scale cavities are readily observed when we consider the hydration free energies of the empty cavities (Figure 5). Up to solutes ∼3.7 Å in radius, the hydration free energy is well described by IGFT within ∼1 *k*_B_*T*. This range of solute sizes is co-incident with the range of solute sizes for which we reliably observe empty solute cavities in water by particle insertion. The hydration free energies of solutes larger than 3.7 Å determined using umbrella sampling, however, are markedly lower than what is predicted by IGFT. Indeed, for the 6.3 Å radius solute IGFT predicts a hydration free energy 64 *k*_B_*T* greater than, or nearly twice, the simulation result. Not accounting for non-Gaussian density fluctuations as the solute size increases subsequently leads to increasingly inaccurate predictions of solute hydration free energies.

### 4.2 Structural Characterization of Cavity Emptying

**Figure 6:**
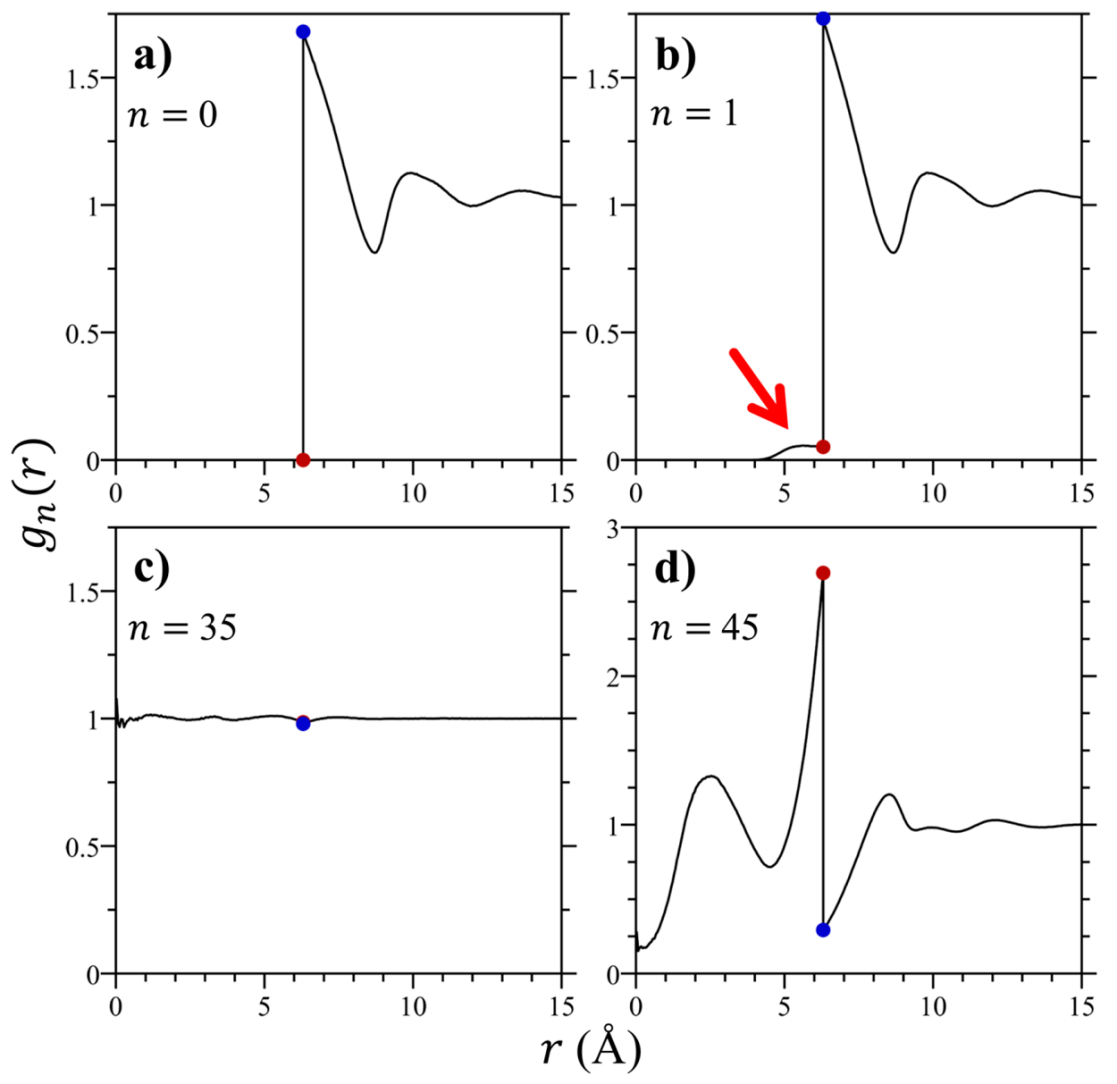
Cavity/water radial distribution functions, *g*_*n*_(*r*), for water occupancies of (a) *n* = 0, (b) *n* = 1, (c) *n* = 35, and (d) *n* = 45 for the 6.3 Å cavity. The blue and red circles in these figures indicates the outer and inner cavity surface contact values of each radial distribution function. The red arrow in panel b indicates the adsorption peak for one water molecule on the inner surface of the cavity.

Insight into the cavity emptying mechanism can be gained by examining the *n* dependent RDFs between the cavity and water. In Figure 6 we report *g*_*n*_(*r*) for *n* = 0, 1, 35, and 45 waters within the 6.3 Å cavity, representative of occupancies from empty to well above ⟨*n*⟩ (RDFs across all cavity occupancy states are reported in Figure S1 in the Supporting Information). The RDF for the empty volume (*g*_0_(*r*), Figure 6a) corresponds to the RDF between a hard sphere solute and the solvent. Water clearly packs around the 6.3 Å hard solute, with a contact peak approximately 60% greater than the bulk solvent density. This contact density is comparable to that observed for similarly sized hard sphere solutes in multiple different representations of water.^15,16,45^ The RDF between water and a cavity with one water molecule within the solute (*g*_1_(*r*), Figure 6b) is nearly the same as that for the empty volume for separations outside the cavity. As anticipated in section 2.1 above, the water density changes discontinuously across the cavity boundary. Within the cavity, we find the lone water tends to adsorb onto the inner surface of the cavity, indicated by the red arrow pointing to the inner packing peak. This water presumably attaches itself to the inner surface to gain attractive interactions with the waters outside the volume that it no longer finds within the emptied solute. The lower value of the packing density of water against the inner wall compared with that against the outer wall indicates that water entry into the cavity is favorable. Similar results are observed for cavities with *n* < ⟨*n*⟩, as seen in Figures S1c–S1ai in the Supporting Information.

When the cavity contains approximately a number of waters close to the mean value (*g*_35_(*r*), Figure 6c) the RDF is practically featureless and nearly equal to 1 across all separations, both inside and outside the cavity. This observation reflects that when averaged overall occupancy states the RDF between the cavity and water is 1 (eq 22) and that the occupancy states closest to ⟨*n*⟩ comprise the dominant contribution to this average. Since the RDF is essentially uniform, it is anticipated that there is little to no barrier for water entry or exit from the cavity when the contact densities inside and outside are nearly equal.

Finally, when the cavity occupancy is significantly greater than that expected by the bulk density, the waters within the volume are expected to pack more like they would in a high pressure solid than in a liquid at ambient conditions. When the cavity contains 45 waters, the density inside the volume is 29% greater than the bulk. In this case the RDF between the cavity and water (*g*_45_(*r*), Figure 6d) exhibits significant structuring. Inside the volume, the waters pack into approximately two bands as indicated by the two peaks in the RDF for *r* < 6.3 Å. The dominant peak inside the cavity is the one pressed against the inner surface. The waters outside the volume, on the other hand, appear to be repelled from its outer surface. This reflects the fact that the waters inside the volume are so tightly packed against the inner wall they push water outside the cavity away, thereby suppressing water’s contact density with the outer wall. The net result of this is that the density of water pressed against the inner wall is greater than that against the outer wall, forming a barrier for water entry into the cavity. Similar results are observed for cavities with *n* > ⟨*n*⟩, as seen in Figures S1ak–S1as in the Supporting Information.

**Figure 7:**
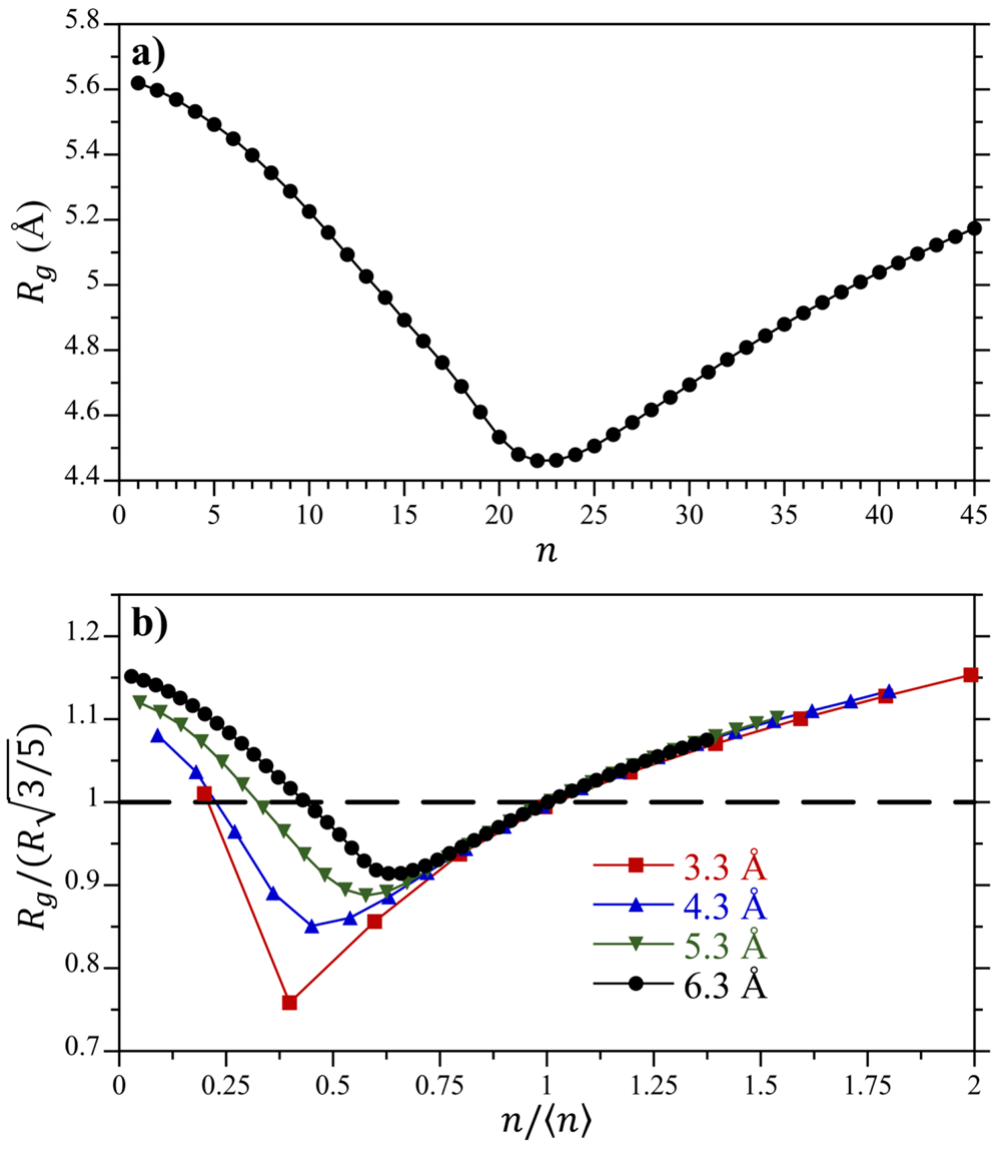
Radius-of-gyration of waters within a cavity as a function of *n* as evaluated by eq 32 at 25 °C and 1 atm determined from simulation. (a) Results for the 6.3 Å radius spherical cavity. (b) Results for the 3.3 Å, 4.3 Å, 5.3 Å, and 6.3 Å radius cavities. The radii-of-gyration in panel b are normalized by the radius-of-gyration of a uniform sphere (), while the occupation numbers are normalized by the mean solvent occupation number (*n*/⟨*n*⟩).

The segregation of the solvent molecule to the inner surface of the cavity as it is emptied can be quantified by considering the radius-of-gyration of the collection of waters inside that volume (Figure 7), evaluated as
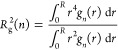
32

For the case of the 6.3 Å cavity, *R*_g_ exhibits a nonmonotonic dependence on *n* (Figure 7a). Beginning near ⟨*n*⟩ (= 34.9 for the 6.3 Å cavity), *R*_g_ decreases with decreasing *n* before reaching a minimum at *n* = 22. This nearly coincides with the observed deviation from the Gaussian behavior of ln *K*_*n*_ in Figure 4. This indicates from *n* = 35 to 22 the waters within the cavity are segregating toward its middle. For values of *n* less than 22, however, this trend reverses, and *R*_g_ increases as water molecules are removed from the cavity (Figure 7a). This effect is such that *R*_g_ for the volume with only 1 water molecule in it is greater than that observed near ⟨*n*⟩, let alone for the largest occupancy state considered. The suggests that over this range of occupancies the water molecules adsorb onto the inner surface of the cavity as it is emptied, facilitating the formation of a bubble within the cavity.

Given that the deviations from Gaussian-like behavior are more pronounced for larger cavities (e.g., Figures 2 and 4), it stands to reason that the nonmonotonic dependence of *R*_g_ on *n* will likewise be more pronounced as well. To facilitate comparison of *R*_g_ across different sized cavities, we use the normalized variables *n*/⟨*n*⟩, corresponding to the fractional water occupancy, and , where  is the radius-of-gyration of a sphere with a uniform density of water inside (i.e., eq 32 with *g*_*n*_(*r*) = 1). In Figure 7b we compare solvent *R*_g_’s for spheres 3.3 4.3, 5.3, and 6.3 Å in radius. For occupancies greater than that for which the minimum in *R*_g_ is observed, each of these cavities appears to follow a universal dependence on *n* using the normalized variables. The values of *n*/⟨*n*⟩ for which *R*_g_ is minimized, on the other hand, depend on the size of the cavity. For the 3.3 Å volume, the minimum occurs when *n* = 2, or *n*/⟨*n*⟩ ≈ 0.40. As the volume gets bigger, the value of *n*/⟨*n*⟩ for which *R*_g_ is minimized shifts to even larger values, occurring near *n*/⟨*n*⟩ ≈ 0.63 for the 6.3 Å volume. This indicates that on the basis of *n*/⟨*n*⟩, the range of fractional occupancy states for which a bubble is stable within the cavity grows as the cavity size increases.

**Figure 8:**
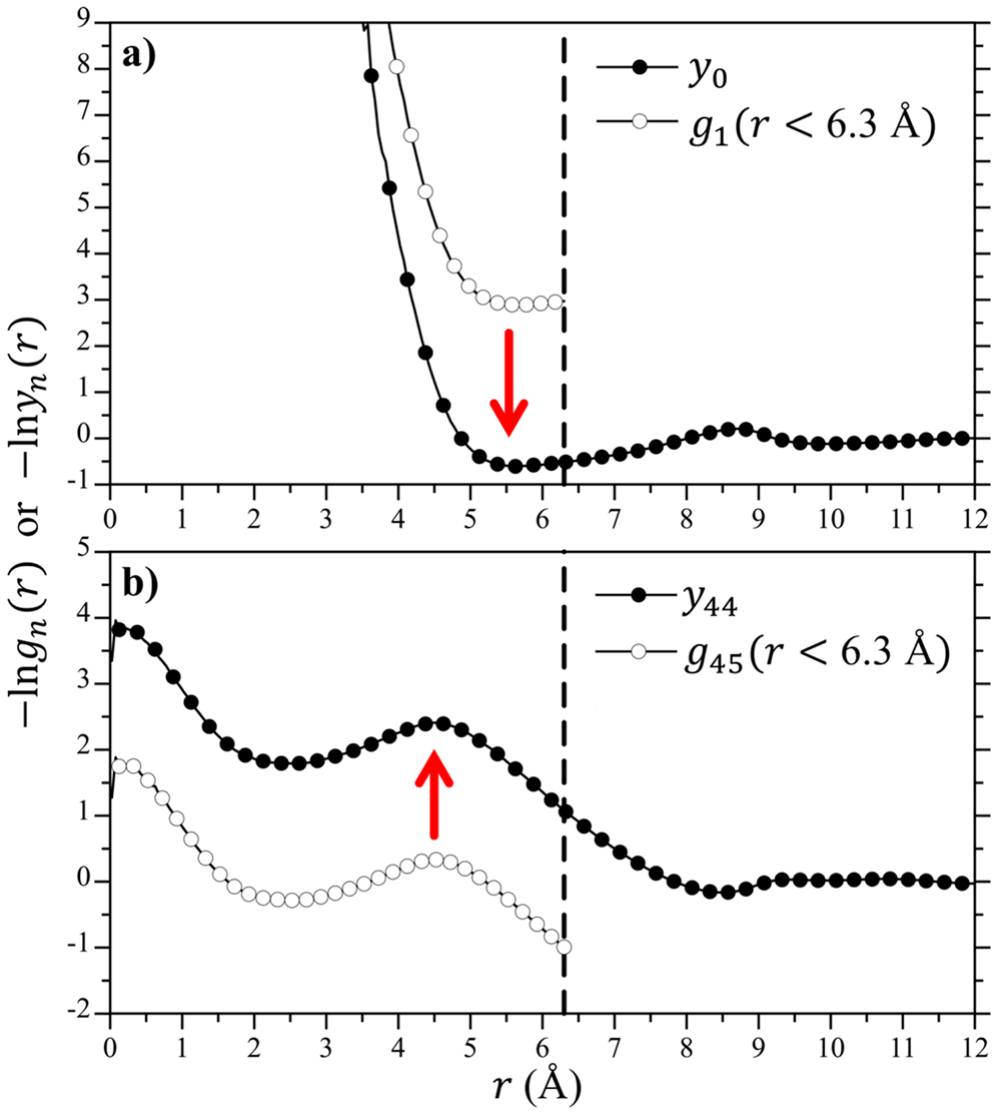
Construction of the cavity correlation function, *y*_*n*_(*r*), from the *g*_*n*_(*r* ≥ *R*) and *g*_*n*__+1_(*r* < *R*) radial distribution functions for the 6.3 Å radius cavity with (a) *n* = 0, and (b) *n* = 44. The red arrows indicate the direction which −ln *g*_*n*__+1_(*r* < *R*) is shifted to meet −ln *g*_*n*_(*r* ≥ *R*) at *R*.

The knitting together of internal and external portions of the RDFs to determine the cavity correlation functions, *y*_*n*_(*r*), for the 6.3 Å sphere is illustrated in Figure 8. Shifting −ln *g*_1_(*r* < 6.3 Å) down by 3.5 to meet −ln *g*_0_(*r* > 6.3 Å) at contact yields a smooth result for −ln *y*_0_(*r*) across all separations (Figure 8a). The resulting downward shift corresponds to a −*k*_B_*T* ln *K*_0_ = −3.5*k*_B_*T* drop in the free energy for adding a single water molecule to an empty cavity. This free energy drop reflects the observed lower value of *g*_1_(*R*^–^) in contact with the inner surface of the cavity (Figure 6b) compared to the great value of *g*_1_(*R*^+^) in contact with the outer surface (Figure 6a). Alternately, shifting −ln *g*_45_(*r* < 6.3 Å) up by 2.1 to meet −ln *g*_44_(*r* > 6.3 Å) at contact yields a smooth result for −ln *y*_44_(*r*) across all separations (Figure 8b). So, in contrast to adding a water molecule to an empty cavity in water, adding a water to an overly packed volume results in an unfavorable −*k*_B_*T* ln *K*_44_ = 2.1*k*_*B*_*T* free energy increase. This, likewise, is reflected by differences in the packing of water against the inner surface versus depletion of waters from the outer surface of the cavity for overly packed states (e.g., Figure 6d).

**Figure 9:**
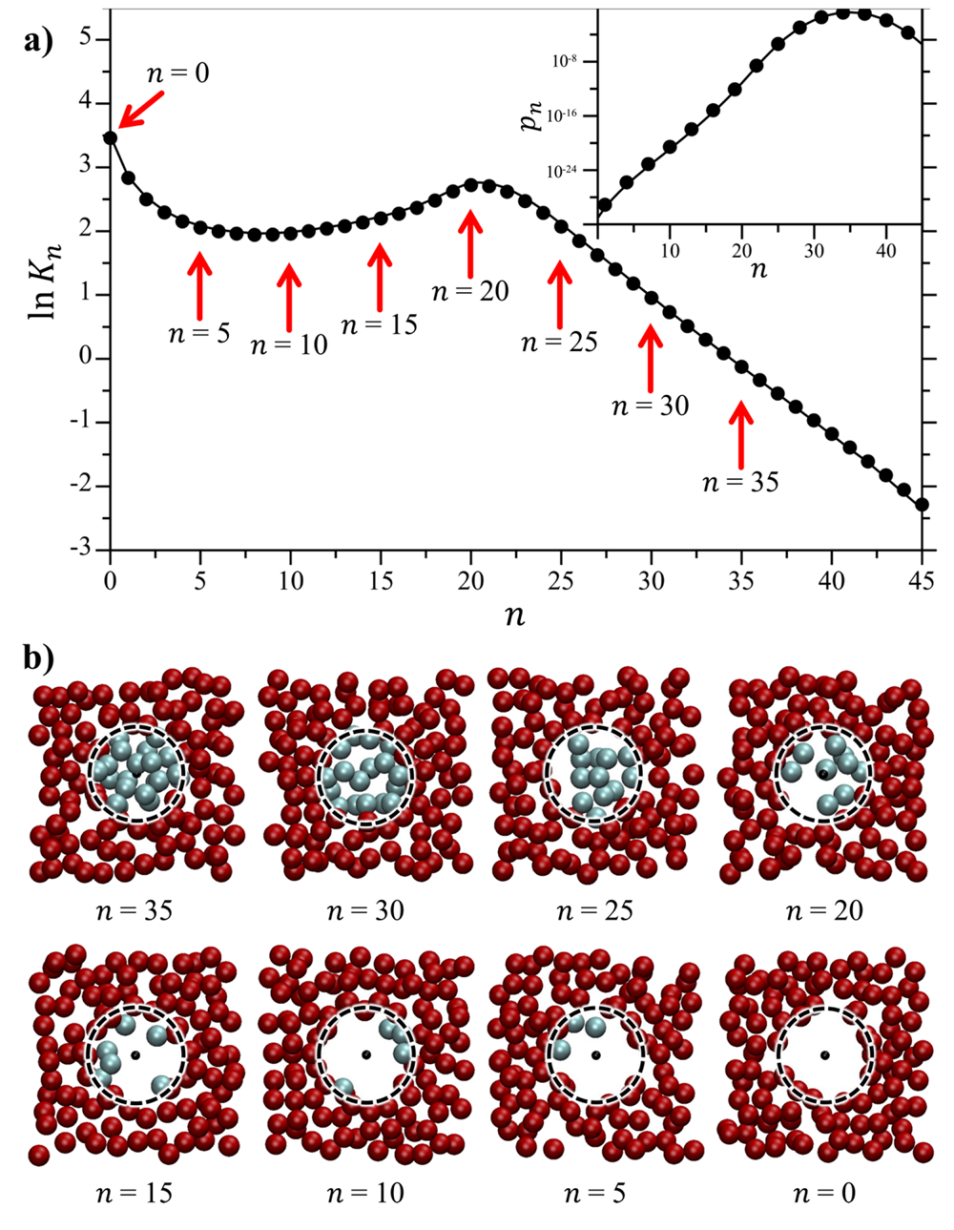
(a) Differential change in the mW water occupational probability, ln *K*_*n*_ = ln(*p*_*n*__+1_/*p*_*n*_),, as a function of *n* for 6.3 Å radius volumes at 25 °C and 1 atm. The solid line indicates the results determined using umbrella sampling, while the points indicate results determined from the cavity radial distribution functions fitted to eq 20. The red arrows indicate the cavity occupancies shown in the simulation snapshots in panel b. The inset figure shows the *p*_*n*_ distribution function determined using umbrella sampling and from the cavity radial distribution functions following eq 21a. The error bars are smaller than the figure symbols. (b) Simulation snapshots of the 6.3 Å cavity taken at cavity occupancies of *n* = 35, 30, 25, 20, 15, 10, 5, and 0. These snapshots are taken from a 4 Å thick slice through the center of the solute cavity to more clearly observe the waters inside. The cavity boundary is indicated by the black dashed circle. The waters outside and inside the cavity are colored red and cyan, respectively. The black dot in the middle of the figure indicates the center of the cavity. These images were rendered using VMD.^53^

In Figure 9a we compare values of ln *K*_*n*_ determined by umbrella sampling against that determined from knitting together the cavity RDFs for the 6.3 Å cavity. (Similarly good results for the 3.3 4.3, and 5.3 Å cavities are reported in Figure S2 in the Supporting Information). The agreement between these two approaches is excellent such that the *p*_*n*_ distributions determined by either technique are indistinguishable (Figure 9a inset). In Figure 9b we show simulation snapshots of slices through the 6.3 Å cavity at *n* values of 35, 30, 25, 20, 15, 10, 5, and 0. In the Gaussian regime (*n* = 35, 30, and 25), the water molecules inside the cavity appear, to a first approximation, uniformly spread across the cavity. A small bubble appears in the lower left side of the *n* = 20 simulation snapshot, just below the water occupancy level for which ln *K*_n_ breaks from the Gaussian prediction. As *n* decreases further, the bubble within the cavity grows even larger until it is completely empty (*n* = 0). For the *n* = 15, 10, and 5 states, the water molecules within the cavity clearly adsorb to the inner surface of the cavity, largely a result of their attraction to the waters in the bulk solvent and consistent with the observed growth of *R*_g_ as the cavity is emptied (Figure 7a).

### 4.3 Theoretical Accounting of Non-Gaussian Density Fluctuations

Here we develop a theoretical framework to modify IGFT to account for non-Gaussian contributions in the description of the solvent density fluctuation within a cavity. Following the observations made above, we expect the onset of non-Gaussian fluctuations to occur over a narrow range of cavity occupancies (e.g., Figure 4), followed by the formation of an empty bubble within the cavity as more waters are removed (e.g., Figure 9). Within the bubble, the remaining waters are adsorbed onto the inner surface of the cavity. These observations form the basis for our extension of the thermodynamic description of cavity emptying.

In the Gaussian fluctuation regime, IGFT predicts that the free energy difference associated with removing a water molecule from a cavity is
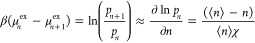
33

Note that ln(*p*_*n*__+1_/*p*_*n*_) = ln *K*_*n*_ is at best an approximation for the derivative of *p*_*n*_ at *n* + 1/2 since the derivative in this expression assumes *n* is continuous rather than discrete. The derivation provided here assumes that *n* is a continuous variable as in the development of IGFT above. This assumption, however, does not impact the outcome of the present derivation.

For large fluctuations that sufficiently reduce the density within the cavity, we hypothesize that a single bubble is nucleated within its boundary. In this case, the thermodynamics of reducing the solvent occupation number is governed by the work against the bulk pressure and the interfacial tension of the bubble. Assuming the bubble can be treated as a spherical void in the cavity carved out from the solvent, the volume and radius of the empty bubble within the cavity are
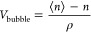
34and
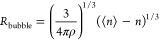
35The surface upon which the interfacial tension acts is not necessarily determined by *R*_bubble_, but rather at an effective radius given as
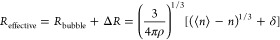
36where Δ*R* = (3/4*πρ*)^1/3^δ is the difference between the effective and actual bubble radii, and is qualitatively similar to a Tolman length.^46^ The resulting effective surface area of the bubble is
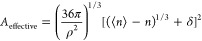
37The free energy associated with creating a cavity with *n* waters and a bubble within its bounds is subsequently determined by the work against the pressure and surface tension as
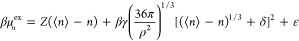
38where *Z* = *βP*/ρ is the compressibility factor, γ is the surface tension of the bubble interface (assumed here to be the vapor/liquid surface tension), and *ε* is a constant that ensures continuity of the free energy between the Gaussian and non-Gaussian regimes. As above, the work associated with removing a single water molecule from the cavity is
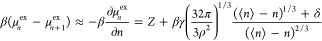
39We propose that a bubble is nucleated inside the cavity when the free energy of removing a water molecule following eq 39, the bubble growth path, is equal to that determined following eq 33, the Gaussian fluctuation path. For states with occupancies less than this transition point, the free energies for removing waters from the cavity following eq 39 are lower than those determined by eq 33. The emptying of the cavity subsequently follows the path for removing water that is lowest in free energy. The occupation number at which the bubble is nucleated, n*, is determined by equating eqs 33 and 39
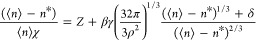
40

A general solution of this expression requires a numerical solution of a quintic equation. Nevertheless, an accurate approximate solution can be derived. Assuming pressure contributions are negligible (*Z* ≈ 0), an excellent approximation near atmospheric pressure, and δ = 0 we find
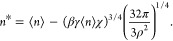
41Expanding the solution of eq 40 in *Z* and δ to second order we find
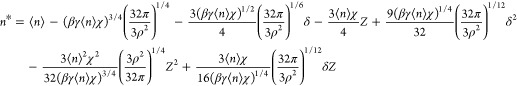
42

For small values of *Z* and δ, such as those found here, use of eq 42 is simpler than the numerical solution of eq 40. Indeed, use of the terms linear in *Z* and δ yields results for the free energy (below) that are practically indistinguishable from the numerical solution of n*. Considering the linear terms in eq 42, it is apparent that n* is always less than ⟨*n*⟩. It is possible to find negative values of n* from eqs 40 and 42. In this case, no bubble is nucleated within the cavity, and density fluctuations are Gaussian over the full range of solvent occupancies.

Ensuring that eq 29a continuously joins with eq 38 at n* to determine *ε*, the free energy of finding *n* waters in the cavity is
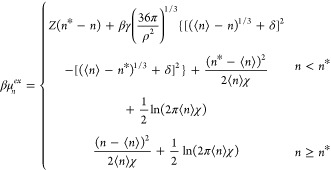
43

Since n* typically is two or more standard deviations less than ⟨*n*⟩, normalization of the *p*_*n*_ (= exp (−βμ_*n*_^*ex*^)) distribution is well approximated by the normalization of the underlying Gaussian distribution. As such, eq 43 is effectively already normalized. The free energy of creating an empty cavity (*n* = 0) is subsequently
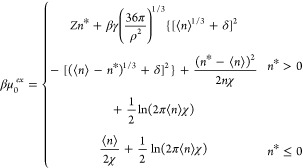
44We refer to this theory as the augmented fluctuation theory (AFT).

We note that AFT draws inspiration from the description of non-Gaussian fluctuations proposed by Huang and Chandler.^28^ The main differences with their proposal and the present derivation are that we introduce a Tolman-like length to the description of interfacial contributions (eq 37), identify the cavity occupancy at which the solvent density fluctuations transition from Gaussian to non-Gaussian (eq. 40), and neglect contributions from the volume accessible to the bubble within the cavity. The first reason we neglect the volume accessibility contribution is that it predicts a divergent hydration free energy for the empty cavity since it assigns a zero probability of observing an empty cavity, i.e., *p*_0_ = 0, due to the reduction of the accessible volume to zero when the bubble volume matches the cavity volume. Second, we hypothesize that the bubble becomes pinned to the center of the cavity as *n* approaches zero, as seen in Figure 9. The reason for this pinning is because for smaller values of *n* either the bubble of volume (⟨*n*⟩ – *n*)/ρ resides entirely within the cavity or a larger bubble that is off center overlaps the cavity so that *n* solvent molecules on one side of the bubble remain in the cavity. Since the probability of observing bubbles even larger than (⟨*n*⟩ – *n*)/ρ becomes negligibly small as the bubble grows, the most likely outcome is that the bubble contained within the cavity is pinned near its center as its occupancy approaches zero.

### 4.4 Comparison of AFT with Molecular Simulation

We fit AFT to the simulation results for βμ_0_^ex^ obtained from simulation using umbrella sampling over the range 2.5 to 6.3 Å (Figure 5). This fitting was conducted using the simulation temperature, pressure, and density of pure mW water. Molinero et al. report a value of 66.0 dyne/cm at 300 K. We subsequently estimate a surface tension of 66.3 dyne/cm at 298.15 K for our fitting using the experimental temperature dependence of the surface tension of water to correct for the slight temperature difference. The fitted value of δ was 0.83, corresponding to a radial increment of Δ*R* = 1.6 Å. We compare the fitted predictions of AFT for the chemical potential of a hard sphere solute in mW water as a function of the solute size against that determined from simulation using umbrella sampling in Figure 5. AFT provides a significantly improved description of the chemical potential over IGFT with quantitative accuracy over the range of solute sizes examined here, giving us confidence in the physics incorporated into the theory.

In Figure 3 we compare simulation results for *p*_*n*_ against the predictions of AFT for the 4.3 5.3, 6.3, and 4.3 Å cavities. Overall, the predictions of AFT are improved against that using IGFT. Notably, AFT accurately captures the fat tail of these distributions. We do observe some differences between the simulations and theory in the case of the 6.3 Å sphere. These differences are clearer if we consider the ln*K*_*n*_ distributions for these cavities (Figure 4). Most significantly, AFT accurately captures the break in *K*_*n*_ associated with the transition from the Gaussian to non-Gaussian regime below n*. For cavity occupancies below n*, however, AFT captures the *n* dependence of ln*K*_*n*_ only semiquantitatively. In particular, the simulation results observe a shallow minimum in ln*K*_*n*_ at intermediate values of *n* between 0 and n*, while AFT predicts only a weak monotonic increase in ln*K*_*n*_ with *n* over this range. We hypothesize that the nonmonotonic dependence of ln*K*_*n*_ over this range is the result of capillary fluctuations at the boundary of the cavity making it harder to remove those final water molecules from the volume, although we have not confirmed this. Nevertheless, AFT accurately threads between the simulation results such that the comparison with the simulation in Figure 3 is significantly improved.

**Figure 10:**
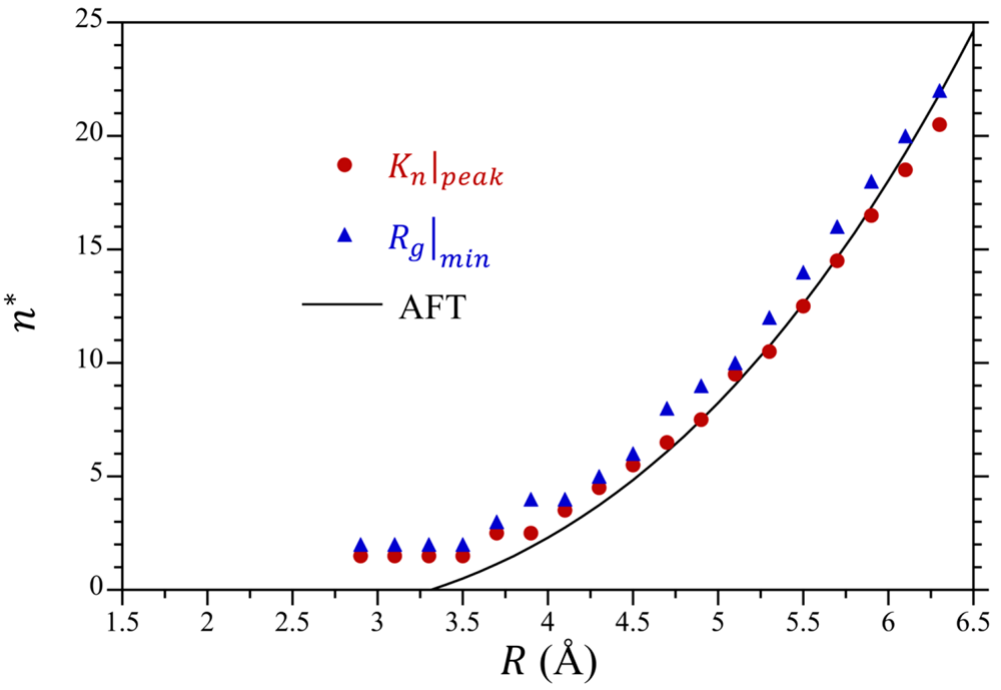
Critical bubble nucleation occupancy, n*, as a function of the cavity radius. Results are reported from the simulations obtained by determination of the peak in ln *K*_*n*_ (e.g., Figure 4), from the minima in the solvent radii-of-gyration (e.g., Figure 7), and AFT following eq 42. The difference between the n* predictions using eqs 40 and 42 are negligible. The figure symbols are defined in the legend.

In Figure 10 we compare the predictions of n* by AFT as a function of the cavity radius against that determined from simulation. We estimated n* from our simulation results in two different ways. The first way, we determined the value of *n* at which ln*K*_*n*_ exhibits a peak, shifted up by 1/2 to better compare the simulation results with the derivative of ln *p*_*n*_ used to evaluate the transition point in AFT (eq 40). The second way we used to evaluate n* from the simulation was to determine the value of *n* at which *R*_g_ is a minimum (Figure 7). As seen in Figure 10, AFT accurately captures the size dependence of n*, especially as the cavity volume grows. This comparison does breakdown for the smallest volumes considered, which is not unexpected given that AFT assumes a continuum treatment of thermodynamics even down to atomic scales. In addition, the agreement between the n* values determined by using either *K*_*n*_ or *R*_g_ is quite good, although the *R*_g_ estimate tends to be slightly larger. This provides strong evidence that the growth in *R*_g_ as the occupation number decreases is directly linked to the onset of non-Gaussian fluctuations in the cavity.

## 5 Conclusions

In this paper we presented a molecular simulation study of the emptying of atomic- and meso-scale cavities in water to get a mechanistic understanding of this process and its relationship to the thermodynamics of hydrophobic hydration. As found in previous studies, we demonstrated, for sufficiently large volumes, the nature of the solvent density fluctuation transitions from Gaussian to non-Gaussian within a solute cavity. The non-Gaussian wing of the distribution as the volume empties is significantly more probable than would be expected if the density fluctuations were normally distributed, which has previously been referred to as a fat-tail distribution. A structural analysis of the waters contained within the cavity found that the occurrence of a fat-tail in the distribution is accompanied by the formation of a bubble within the cavity. A statistical thermodynamic analysis of solvent packing on either side of the boundary between the cavity’s interior and the bulk solvent demonstrated that the formation of the bubble results in the net adsorption of water onto the cavity’s inner surface, reducing the penalty for removing waters from the cavity, thereby fattening the distribution.

The finding that the formation of a bubble within the cavity occurs over a narrow range of occupancy states led us to propose an empirical correction to a theory we previously developed to account for Gaussian solvent density fluctuations within solute cavities, IGFT. This augmented fluctuation theory, or AFT, accounts for the formation of a bubble by smoothly joining IGFT with a macroscopic thermodynamic description of a bubble’s interface at the transition occupancy. AFT successfully describes the free energies of hard sphere solute hydration over a much broader range of solute size scales than IGFT, capturing the fat-tail distribution and predicting the cavity occupancies at which the bubble forms. We note that while we only considered solutes up to 6.3 Å in radius in a coarse-grained representation of water, we previously reported simulations of a more realistic description of water with solutes up to 18 Å in radius where it was demonstrated that a nascent version of AFT accurately describes bubble formation in this system.^32^ This lends confidence to the accuracy of the theory presented here over a wide range of cavity sizes.

The statistical thermodynamic framework introduced here connecting the *p*_*n*_ distribution to solvent packing both inside and outside a solute cavity provides a new route for both evaluating solvent density fluctuations within the volume and interpreting their origin. We demonstrated here that the transition from Gaussian to non-Gaussian-like behavior in the emptying of a cavity is accompanied with the adsorption of water molecules inside the volume onto the inner surface of the cavity. As a result of this adsorption and accompanying bubble formation, the penalty for removing waters from the cavity is significantly lower than that would be anticipated assuming Gaussian fluctuations. Within the context of this statistical thermodynamic framework, it is worthwhile to consider other reasons large density fluctuations can deviate from Gaussian behavior. Notably, simulations of alternate solvents that include dense hard spheres,^47−49^ an isotropic model reproducing water’s RDF,^50^ the Jagla model,^51^ and a water-like model with weakened hydrogen-bonds^51^ found that solvent density fluctuations within cavities appear Gaussian near ⟨*n*⟩ but exhibit significantly suppressed occupation probabilities as *n*→ 0. This would be indicated by large positive deviations from the linear Gaussian response on a ln *K*_*n*_ plot, in contrast to the negative deviations observed here. Compared to water these solvents are more repulsive to one another. For example, the isotropic water model noted above has a pressure of 7500 bar at 1 g/cm^3^ at 25 °C.^50^ Rather than adsorb onto the inner surface of the cavity to gain attractive interactions with the bulk solvent, we anticipate that these repulsive solvents would be pushed from the inner surface to positions deeper inside the cavity. As such, we would expect the inner surface adsorption peak observed in Figure 6b to migrate toward the center of the cavity with only a minimal contact density at the inner surface. This would establish a more significant barrier for removing those solvent molecules from the cavity, suppressing larger density fluctuations, in accordance with the simulation observations.

The present theory does not account for contributions such as capillary fluctuations at the bubble interface that can make it harder to fully empty the cavity. In addition, for nonspherical volumes additional contributions associated with Gaussian curvature and higher order corrections may also have to be taken into account.^52^ Nevertheless, we expect the present description will still capture the onset of non-Gaussian solvent density fluctuations even in the case of nonspherical cavities since we expect the initial bubble will be spherical, only adopting its final nonspherical shape as the cavity is emptied.

## Supporting Information Available

Additional information on (1) the full set of *g*_*n*_(*r*) distributions about the 6.3 Å cavity in water; and (2) ln *K*_*n*_ as a function of *n* for the 3.3 Å, 4.3 Å, and 5.3 Å cavities in water determined using umbrella sampling and from the inner and outer water contact densities (PDF)
