# Supplementary material for: Gaussian and Non-Gaussian Solvent Density Fluctuations within Solute Cavities in a Water-like Solvent
Source: J Chem Theory Comput. 2023 Jul 12;20(4):1505–18. doi: 10.1021/acs.jctc.3c00387 (PMC10902835; doi:10.1021/acs.jctc.3c00387)
Supplement: Supplementary file 1 — ct3c00387_si_001.pdf [file ct3c00387_si_001.pdf]

Supporting Information for

Gaussian and Non-Gaussian Solvent Density Fluctuations within Solute Cavities in a Water-like Solvent

Henry S. Ashbaugh

Tulane University, Chemical and Biomolecular Engineering, New Orleans, LA 70118

In this supplement we provide additional information regarding the hydration of cavities in water. Figures S1a – S1at (pages S2 – S13) report the full $g_n(r)$ distributions about the 6.3 Å cavity in water for $n =$ 0 to 45. Figure S2 (page S14) reports the values of $\ln K_n$ as a function of n for the 3.3 Å, 4.3 Å, and 5.3 Å cavities in water determined using umbrella sampling and from the inner and outer water contact densities determined from their cavity-water radial distribution functions.

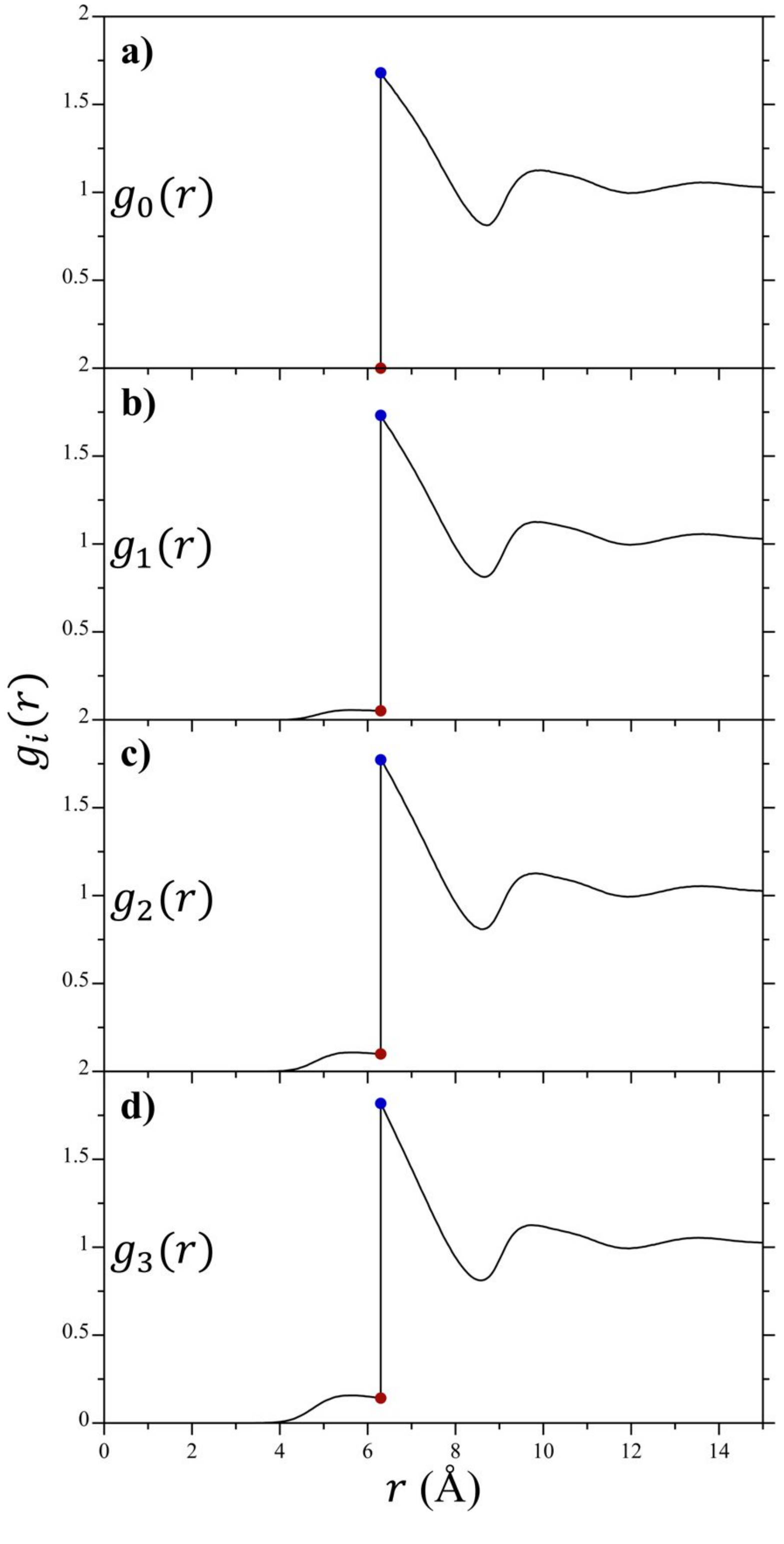

Figures S1a – S1d. Cavity-water radial distribution functions for the $R = 6.3$ Å cavity at 25°C and 1 atm for $g_0(r)$ to $g_3(r)$ The radial distribution functions are indicated by the thin solid line. The inner and outer cavity boundary contact values are indicated by the red and blue circles, respectively.

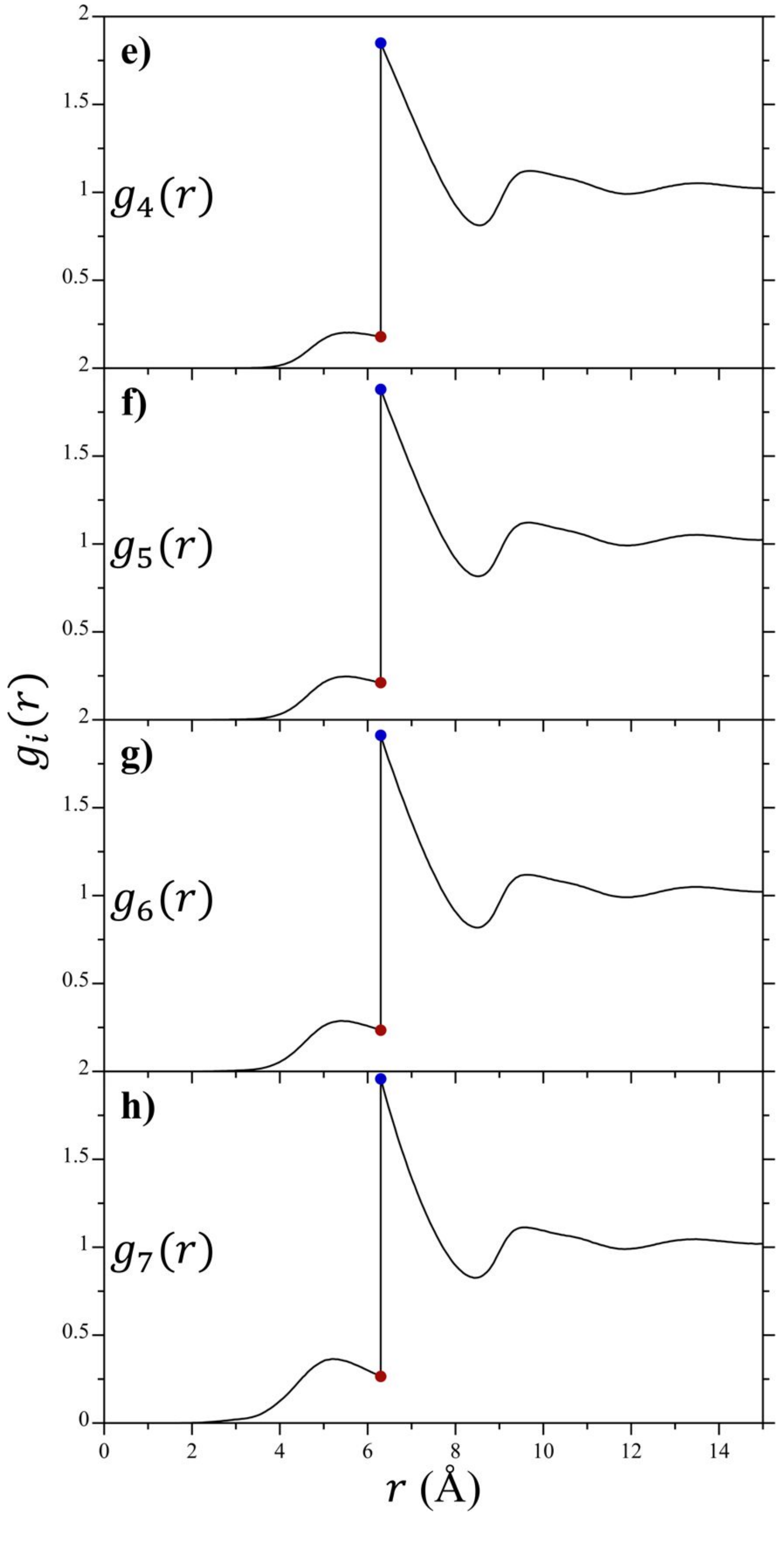

Figures S1e – S1h. Cavity-water radial distribution functions for the $R =$ 6.3 Å cavity at 25°C and 1 atm for $g_4(r)$ to $g_7(r)$ The radial distribution functions are indicated by the thin solid line. The inner and outer cavity boundary contact values are indicated by the red and blue circles, respectively.

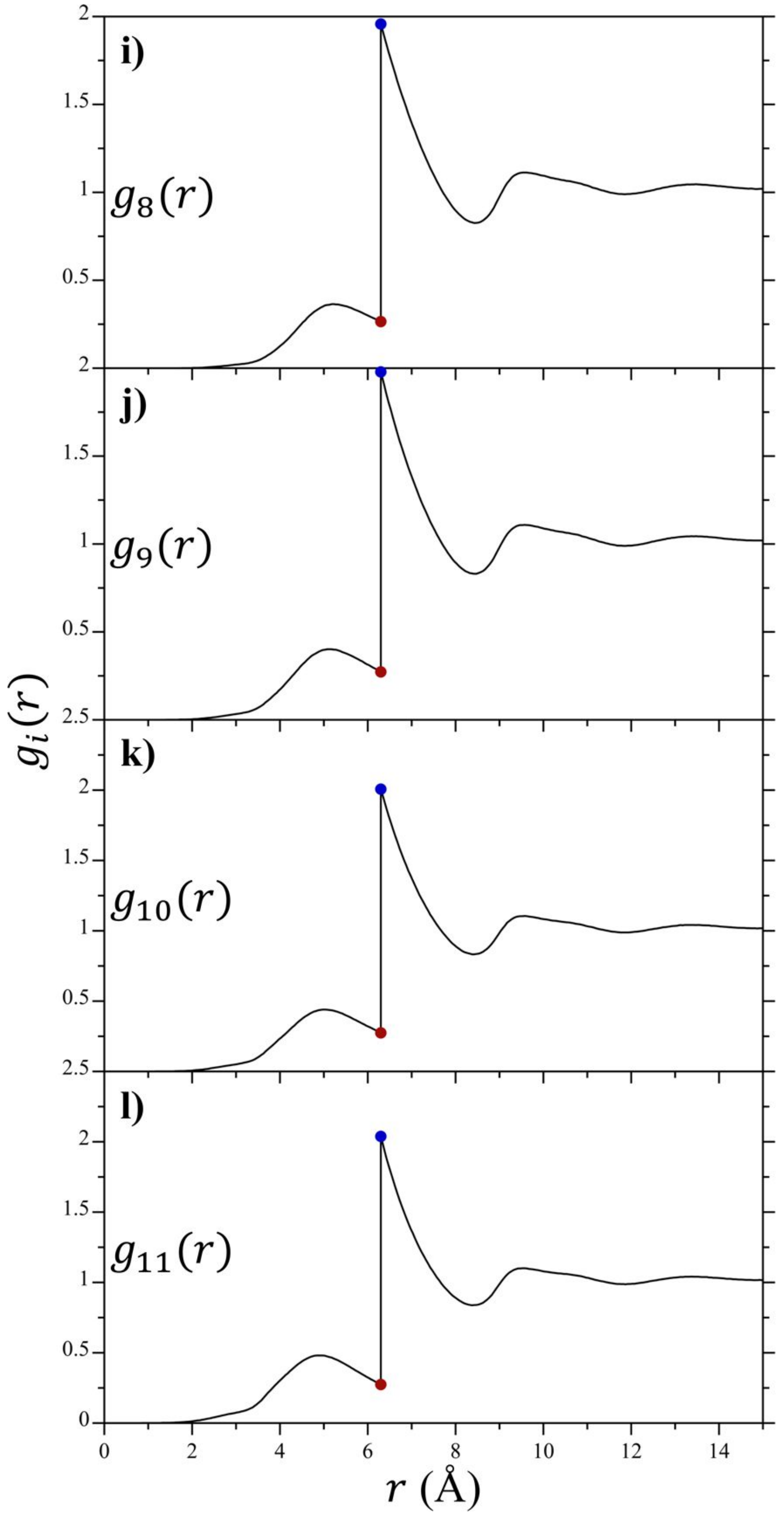

Figures S1i – S1l. Cavity-water radial distribution functions for the $R = 6.3$ Å cavity at 25°C and 1 atm for $g_8(r)$ to $g_{11}(r)$ The radial distribution functions are indicated by the thin solid line. The inner and outer cavity boundary contact values are indicated by the red and blue circles, respectively.

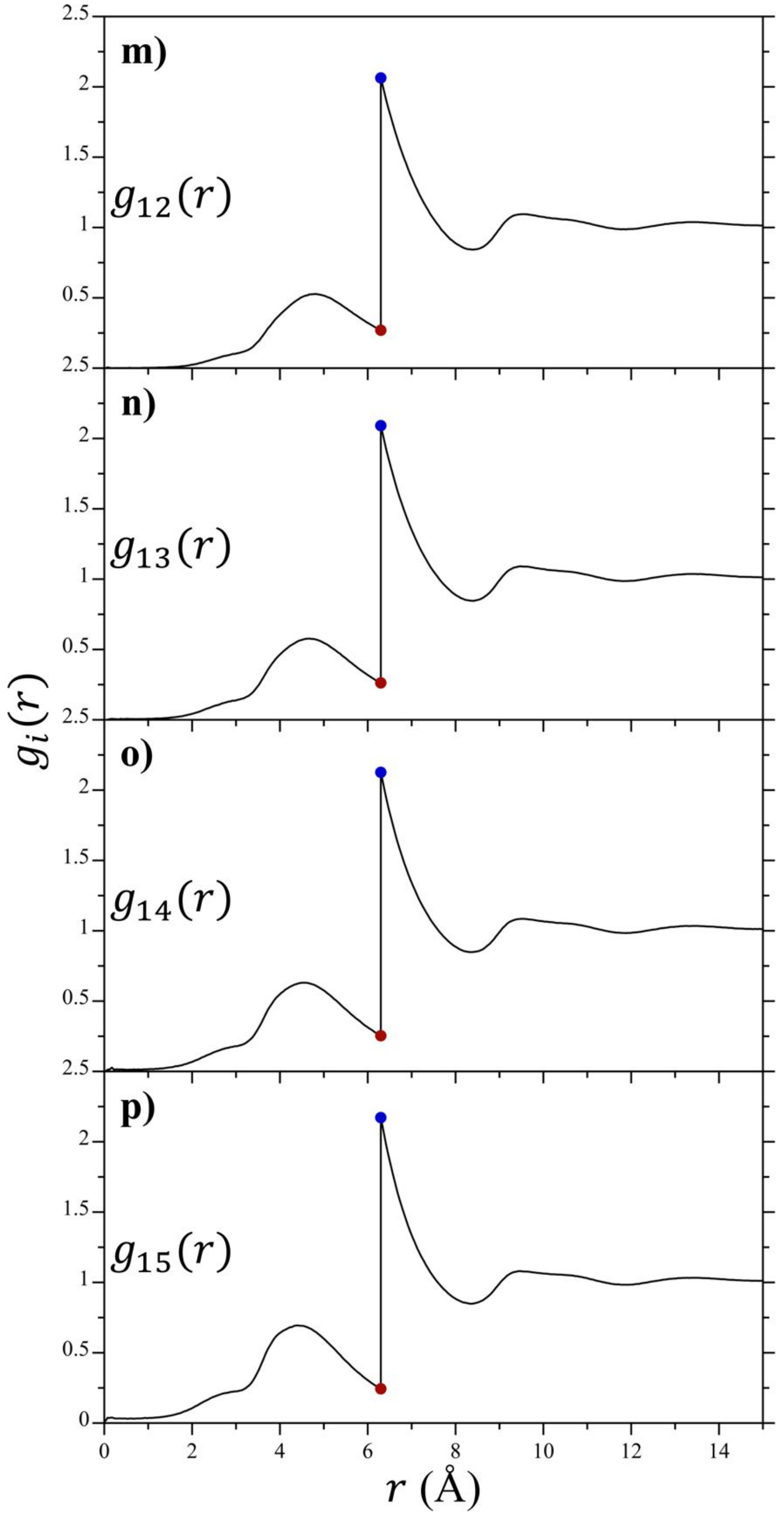

Figures S1m – S1p. Cavity-water radial distribution functions for the $R =$ 6.3 Å cavity at 25°C and 1 atm for $g_{12}(r)$ to $g_{15}(r)$ The radial distribution functions are indicated by the thin solid line. The inner and outer cavity boundary contact values are indicated by the red and blue circles, respectively.

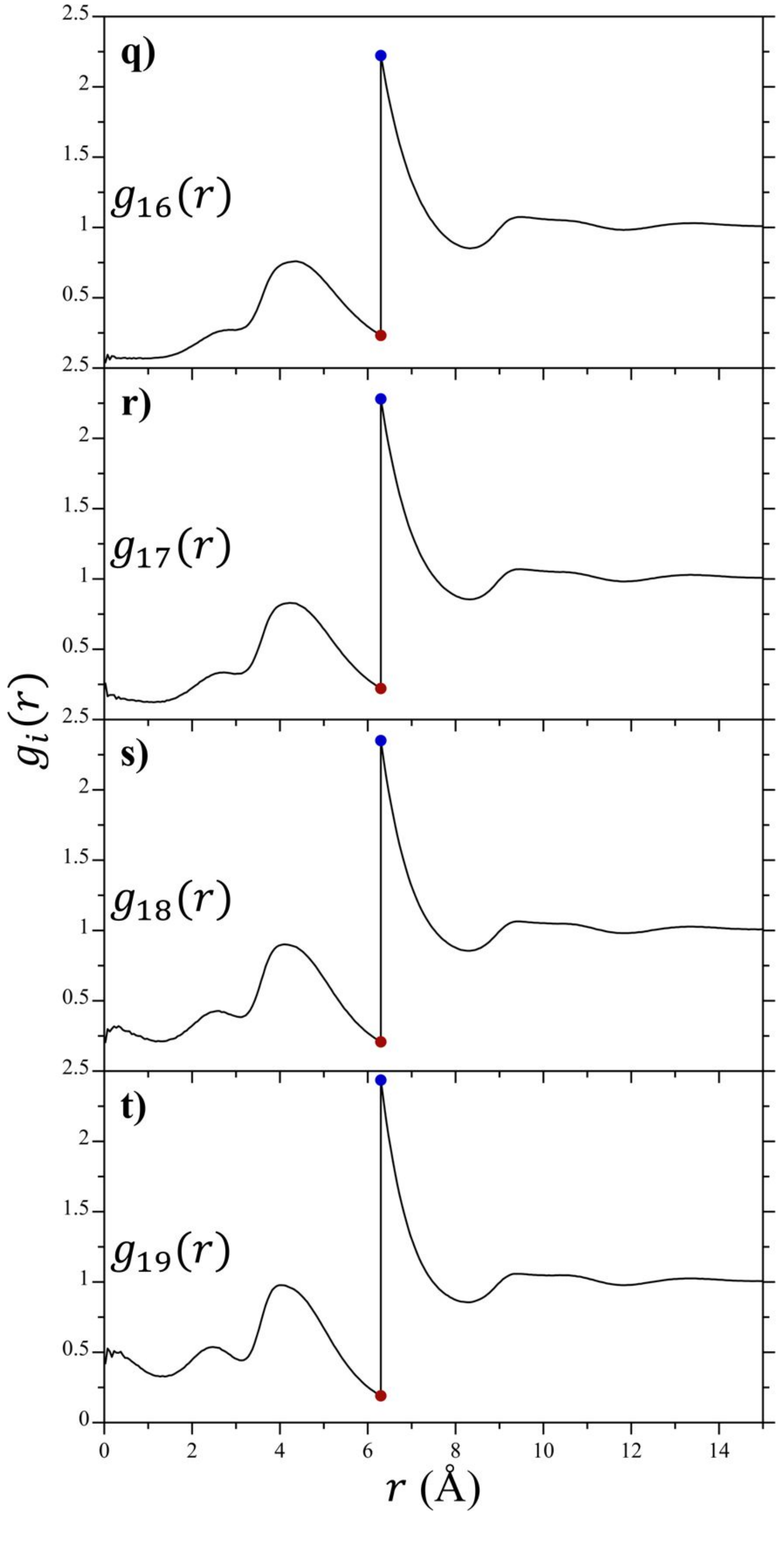

Figures S1q – S1t. Cavity-water radial distribution functions for the $R =$ 6.3 Å cavity at 25°C and 1 atm for $g_{16}(r)$ to $g_{19}(r)$ The radial distribution functions are indicated by the thin solid line. The inner and outer cavity boundary contact values are indicated by the red and blue circles, respectively.

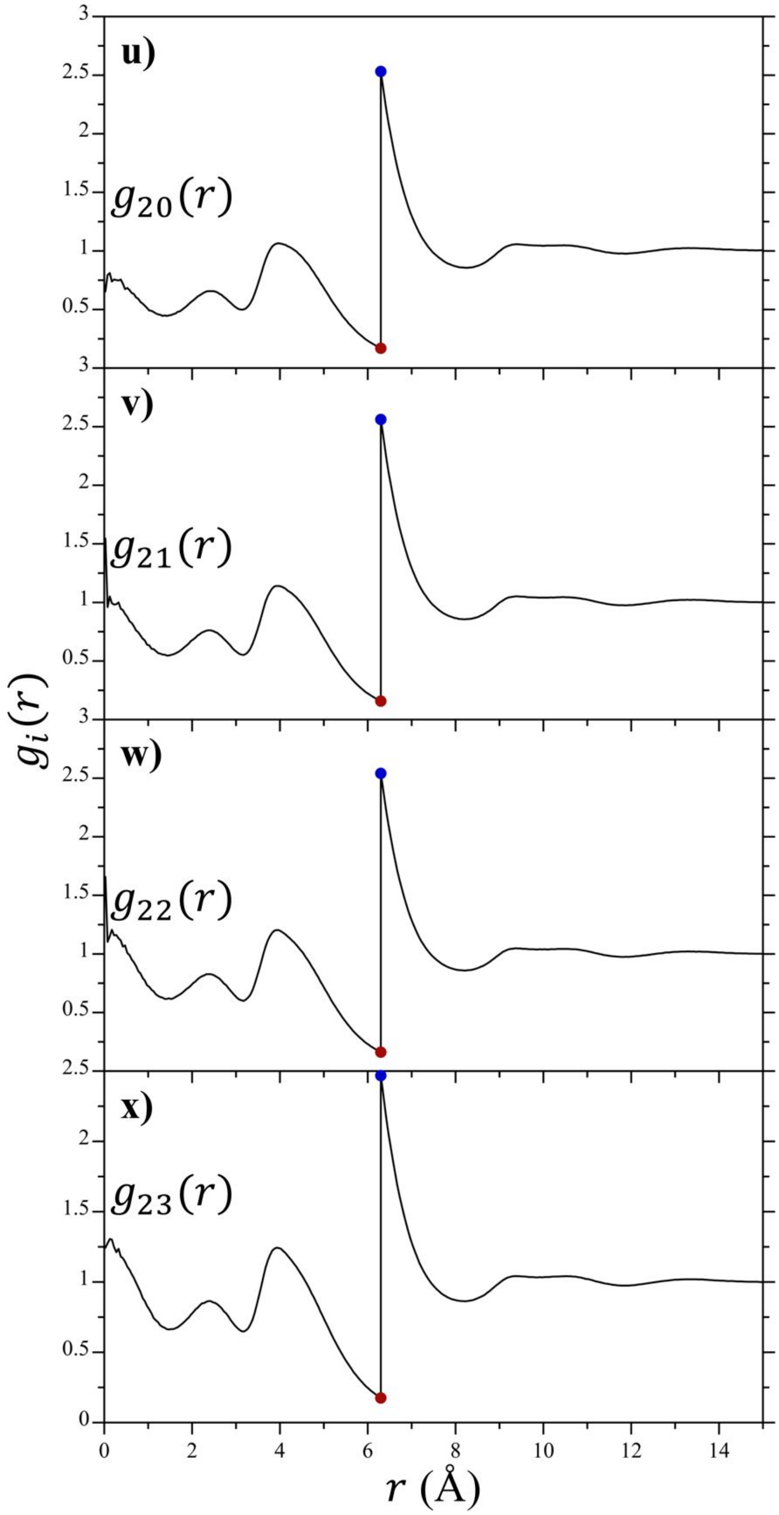

Figures S1u – S1x. Cavity-water radial distribution functions for the $R =$ 6.3 Å cavity at 25°C and 1 atm for $g_{20}(r)$ to $g_{23}(r)$ The radial distribution functions are indicated by the thin solid line. The inner and outer cavity boundary contact values are indicated by the red and blue circles, respectively.

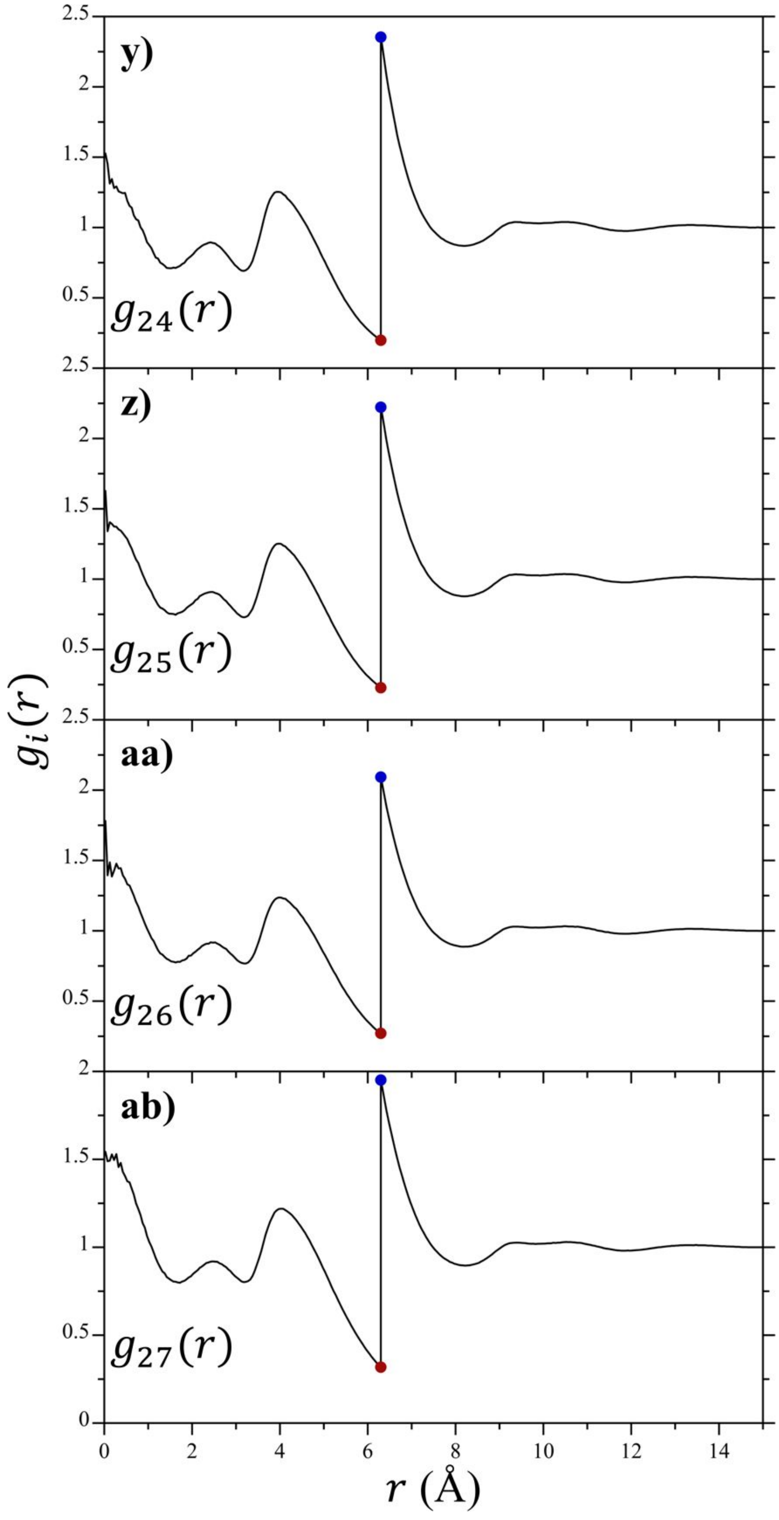

Figures S1y – S1ab. Cavity-water radial distribution functions for the $R =$ 6.3 Å cavity at 25°C and 1 atm for $g_{24}(r)$ to $g_{27}(r)$ The radial distribution functions are indicated by the thin solid line. The inner and outer cavity boundary contact values are indicated by the red and blue circles, respectively.

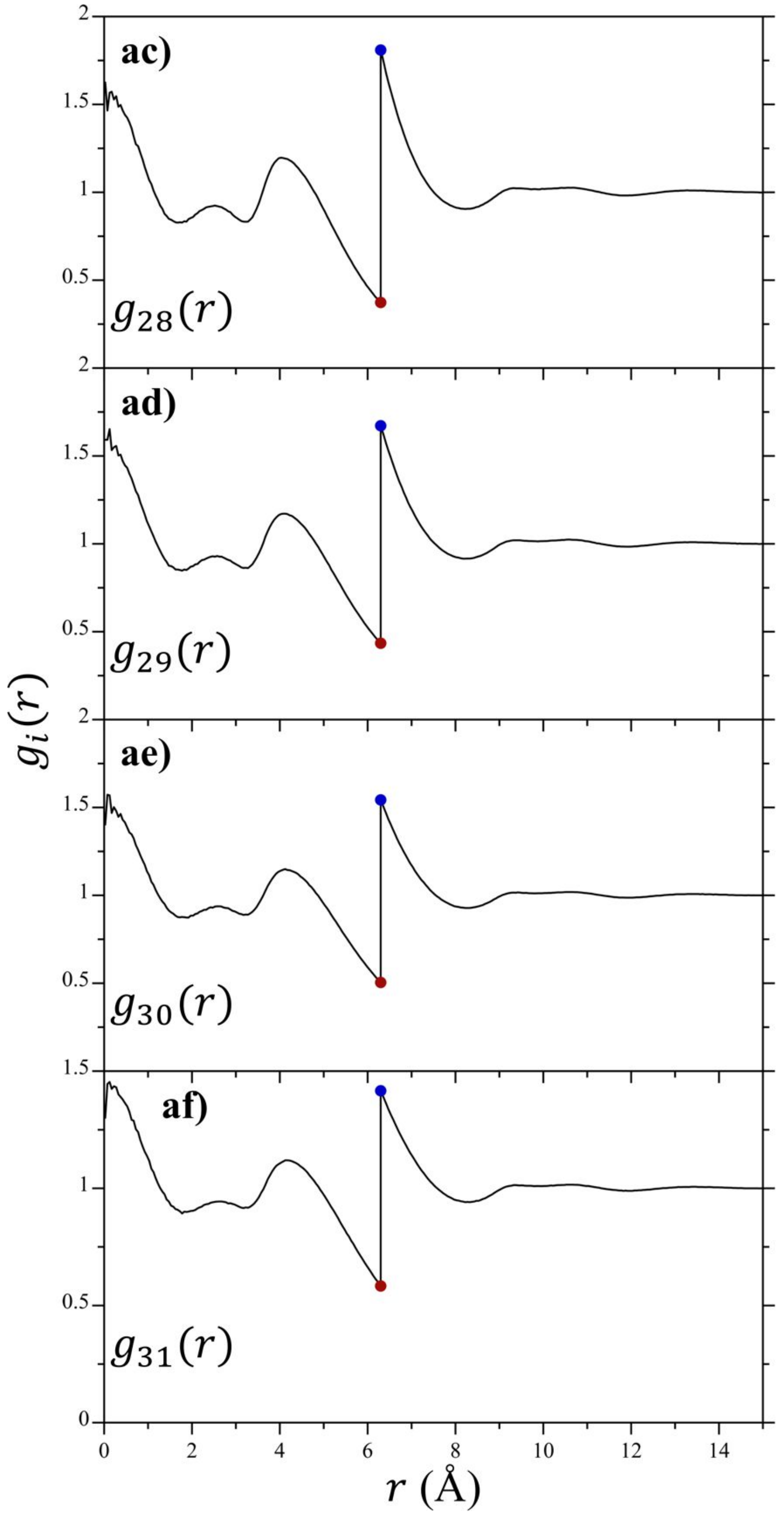

Figures S1ac – S1af. Cavity-water radial distribution functions for the $R =$ 6.3 Å cavity at 25°C and 1 atm for $g_{28}(r)$ to $g_{31}(r)$ The radial distribution functions are indicated by the thin solid line. The inner and outer cavity boundary contact values are indicated by the red and blue circles, respectively.

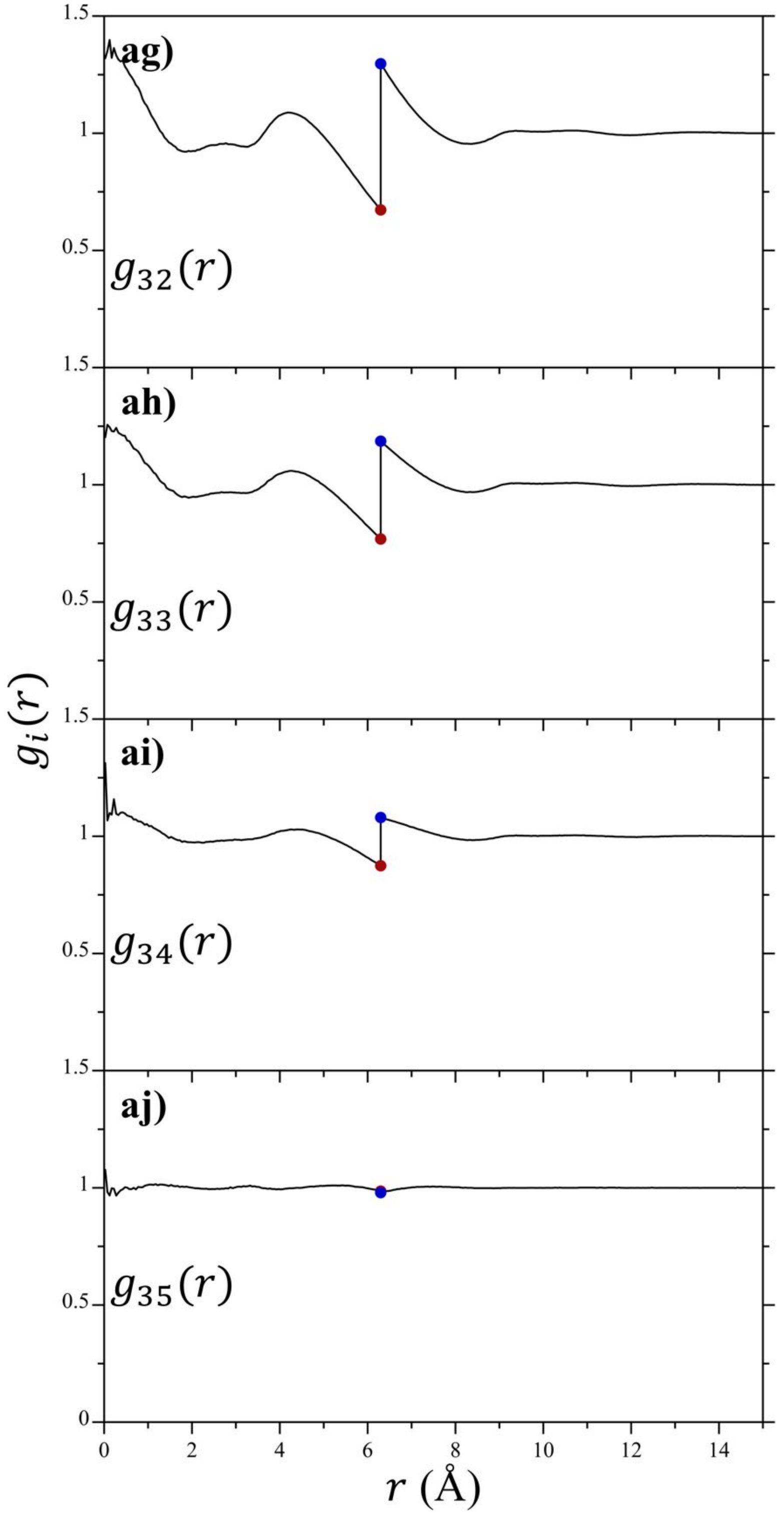

Figures S1ag – S1aj. Cavity-water radial distribution functions for the $R =$ 6.3 Å cavity at 25°C and 1 atm for $g_{32}(r)$ to $g_{35}(r)$ The radial distribution functions are indicated by the thin solid line. The inner and outer cavity boundary contact values are indicated by the red and blue circles, respectively.

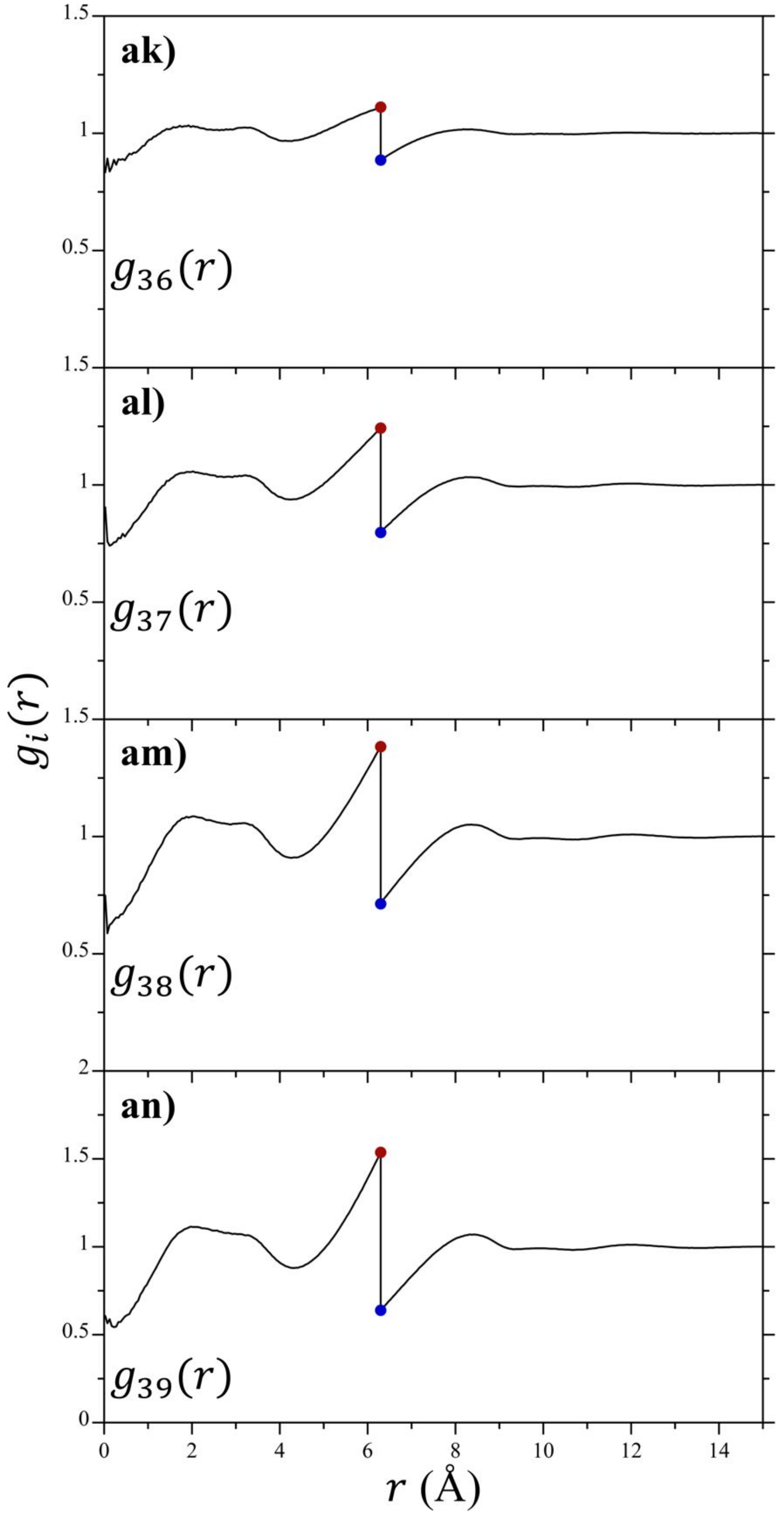

Figures S1ak – S1an. Cavity-water radial distribution functions for the $R =$ 6.3 Å cavity at 25°C and 1 atm for $g_{36}(r)$ to $g_{39}(r)$ The radial distribution functions are indicated by the thin solid line. The inner and outer cavity boundary contact values are indicated by the red and blue circles, respectively.

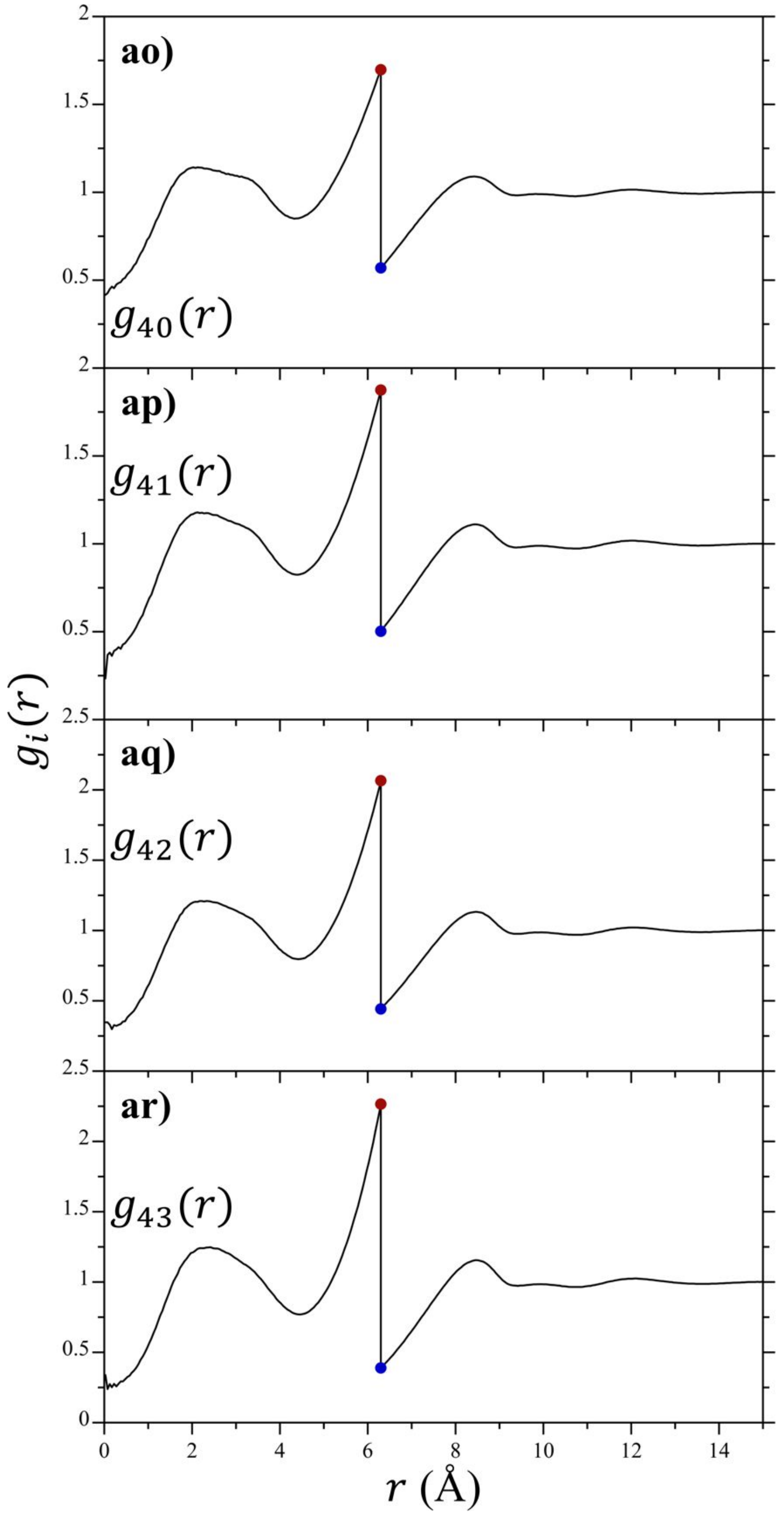

Figures S1ao – S1ar. Cavity-water radial distribution functions for the $R =$ 6.3 Å cavity at 25°C and 1 atm for $g_{40}(r)$ to $g_{43}(r)$ The radial distribution functions are indicated by the thin solid line. The inner and outer cavity boundary contact values are indicated by the red and blue circles, respectively.

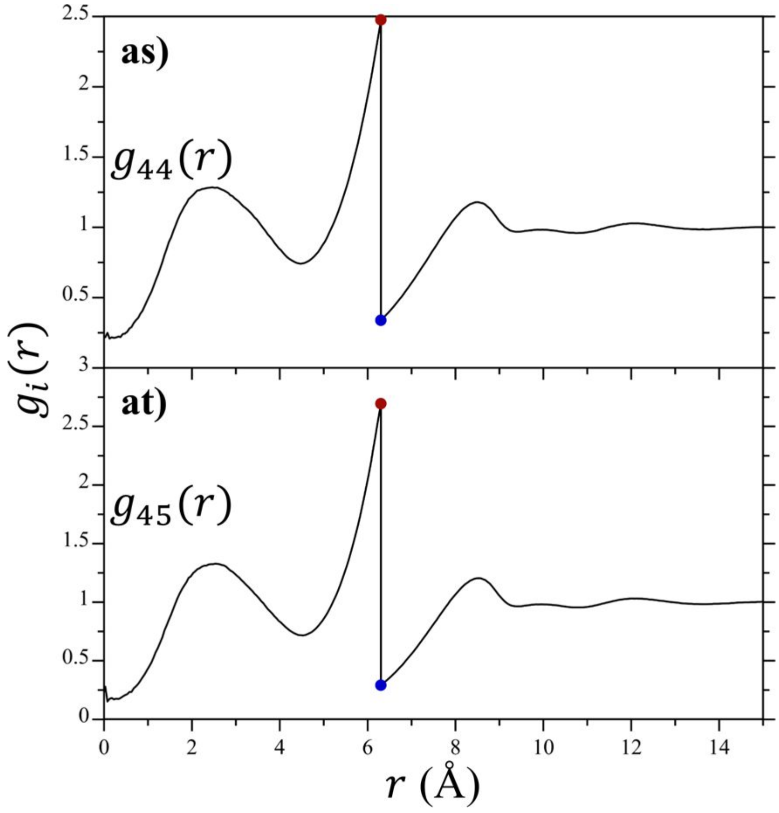

Figures S1as – S1at. Cavity-water radial distribution functions for the $R =$ 6.3 Å cavity at 25°C and 1 atm for $g_{44}(r)$ to $g_{45}(r)$ The radial distribution functions are indicated by the thin solid line. The inner and outer cavity boundary contact values are indicated by the red and blue circles, respectively.

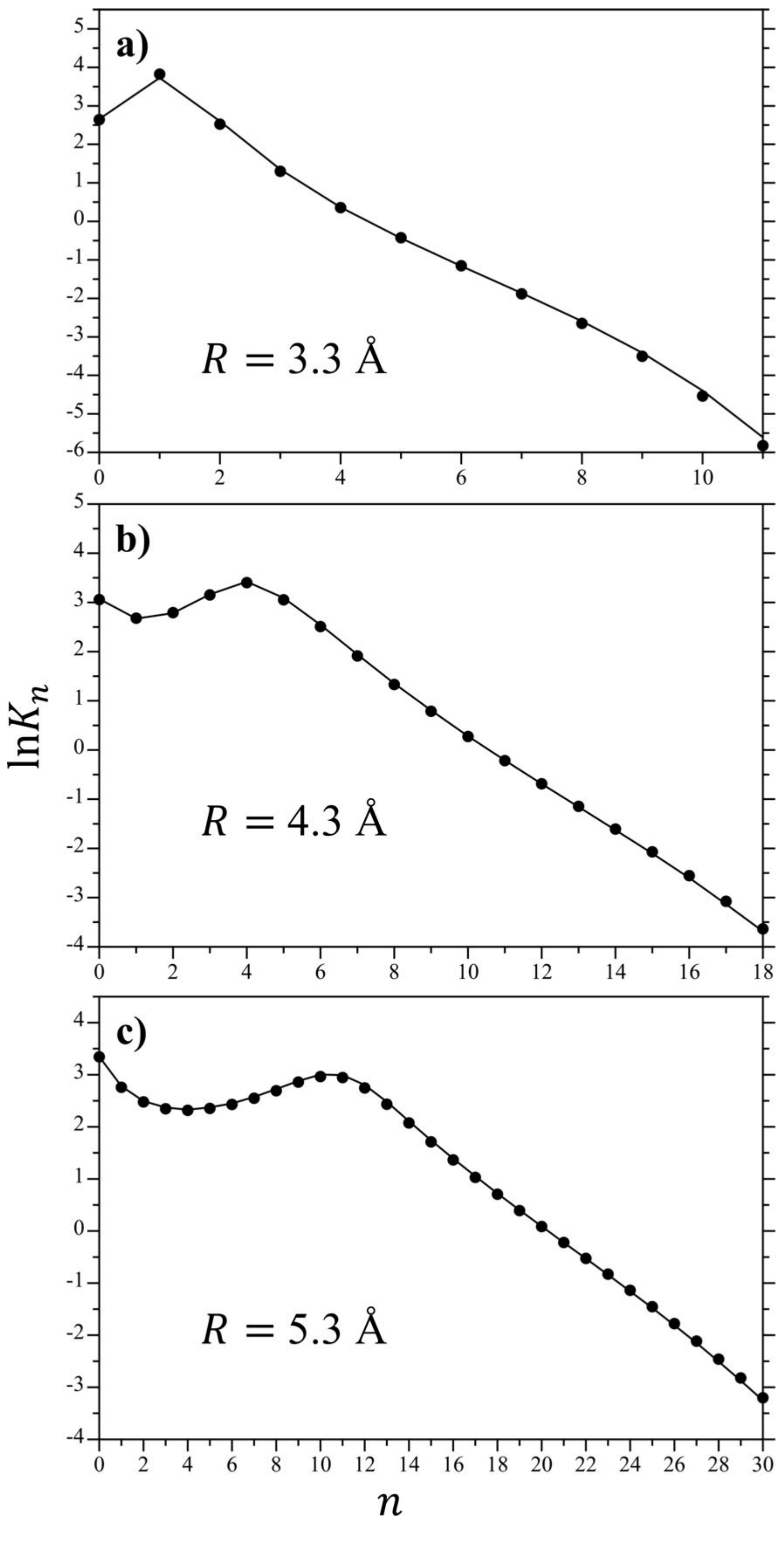

Figure S2. Differential change in the water occupational probability, $\ln K_n = \ln(p_{n+1}/p_n)$ for the 3.3 Å (**a**), 4.3 Å (**b**), and 5.3 Å (**c**) radius cavities at 25°C and 1 atm. The lines indicate result determined using umbrella sampling, while the points indicate results determined from the cavity radial distribution functions fitted to eq. (20).
